# Wildlife health in the One Health era: lessons from a 200-year institutional case study

**DOI:** 10.1098/rspb.2026.0285

**Published:** 2026-09-16

**Authors:** Andrés Valenzuela-Sánchez, Javier Sánchez Romano, Becki Lawson, Katharina Seilern-Macpherson, Robert Deaville, Rosie S. Williams, Rosie Woodroffe, Louise Gibson, Amanda Guthrie, Simon Spiro, María Díez-León, Stuart Patterson, Benjamin Tapley, Sarah Haddow, Andrew A. Cunningham

**Affiliations:** ^1^ Institute of Zoology, Zoological Society of London, UK; ^2^ ONG Ranita de Darwin, Chile; ^3^ Zoological Society of London, UK; ^4^ Royal Veterinary College, University of London, UK

**Keywords:** wildlife health, disease ecology, conservation, wildlife management, One Health, wild animal, pandemic, disease, pathogen, welfare

## Abstract

The One Health framework has drawn increasing attention to the interdependence of human, animal and environmental health. In this context, wildlife health has become more visible, yet its role remains inconsistently defined and is often framed primarily in terms of wildlife as a source of pathogens of concern to humans and domestic animals. This perspective reflects only a limited subset of the reasons why different sectors of society value healthy wildlife. Marking the 200-year anniversary of the Zoological Society of London (ZSL), a prominent institution in the emergence and development of wildlife health as a field, we synthesize historical, conceptual and applied perspectives to clarify what wildlife health means in practice and how it has been studied and managed over time. Our analyses show that wildlife health research remains dominated by disease-centric framings, with limited engagement with health as a state of comprehensive well-being. Using the Zoological Society of London as an institutional case study and drawing on six applied examples, we illustrate how wildlife health research can be embedded in policy and practice. We conclude by outlining key priorities for advancing wildlife health within the One Health era.

## 1. Introduction

The increasing recognition of interdependencies among humans, domestic animals, wildlife and ecosystems has driven interest in integrative health approaches beyond single-species perspectives, crystallizing in the One Health framework, which seeks to jointly promote the health of people, animals and the environment [1,2]. The growing prominence of One Health is evident not only in the scientific literature but also across academic institutions and funding landscapes, and in global governance. This is exemplified by the establishment of the One Health High-Level Expert Panel in 2021—mandated by the World Health Organization (WHO), the Food and Agriculture Organization of the United Nations, the World Organisation for Animal Health (WOAH) and the United Nations Environment Programme [2]—and by the inclusion of an explicit One Health article in the WHO Pandemic Agreement adopted in 2025, formally committing Parties to integrated human, animal and environmental health approaches in pandemic prevention and preparedness [3].

Within this expanding societal interest in One Health, wildlife health has become increasingly visible, yet scientists and practitioners have raised concerns that wild animals in One Health contexts are often framed primarily in terms of pathogen risks to humans and domestic animals [4–6], while the ecological dimensions of One Health remain comparatively underdeveloped [7]. Such framings reflect only a limited subset of the reasons why different sectors of society value healthy wildlife (here, we use the definition of wildlife as referring to wild animals, reflecting the traditional usage of the term within wildlife health). For some, wildlife holds intrinsic importance; for others, it plays key roles in subsistence and recreational hunting, cultural practices and human well-being [5]. Wildlife also underpins ecosystem functioning, contributing to processes such as nutrient cycling, trophic regulation and ecosystem resilience, with implications for human health through pathways that extend well beyond infectious disease alone [8]. This tension is exemplified in the strategic vision of the WOAH's Wildlife Health Framework, which promises to ‘guide Members in their use of One Health strategies at national level to help manage the risk of disease emergence at the human-animal-ecosystem interface, while uplifting the value of wildlife, and the need to protect, rather than vilify, wildlife in disease emergence scenarios’ [9].

Given this situation, a timely synthesis is needed to clarify what wildlife health means in practice, how it has been studied and managed over time, how the field has evolved within an increasingly integrative health landscape and what directions its future development might take. We combine this broad conceptual aim with an institutional case-study focus on the Zoological Society of London (ZSL), the world's oldest scientific zoological society. This focus is partly shaped by the context of this Special Feature, which marks ZSL's 200-year anniversary, but it also provides a rare opportunity to examine how wildlife health has developed within a long-standing institution.

ZSL has played a prominent and sustained role in the formation of wildlife health as a field of practice. This role can be examined from a historically informed institutional perspective because of ZSL's long-standing scientific infrastructure, including the Prince Philip Zoological Library and Archives, founded in 1826, and its early scientific publishing activity, including the *Proceedings of the Zoological Society of London*, first issued in 1830 and later continued as the *Journal of Zoology* [10,11]. Such conditions are unlikely to be easily replicated across many institutions, making this synthesis valuable as a source of broader lessons for wildlife health. Using ZSL as an institutional case study, we ask how wildlife health research has taken shape within this organization, and how it has been embedded in policy and practice. By combining a broad synthesis of wildlife health with an institutional case study, we aim to distil insights that may be informative for those working in wildlife health in the One Health era.

## 2. What is wildlife health?

The concept of health has been defined in diverse ways, ranging from negative formulations, such as the absence of disease, to positive formulations that emphasize well-being [12,13]. In human health, positive definitions have been particularly influential, most notably the definition adopted in the Constitution of the World Health Organization in 1948, which conceptualized health as a state of comprehensive physical, mental and social well-being rather than merely the absence of disease [13] (table 1). This philosophical orientation has also informed commonly used definitions of animal health (table 1).

**Table 1.** Definitions of the concept of wildlife health and commonly used definitions of human and animal health.

concept	definition	source
wildlife health	the absence of disease.	[5]
	capacity derived from interacting individual, environmental and social factors that allows the individual or population to cope with all demands of daily life.	[5]
	social construct where individuals or population meet our social and scientific expectations for how they exist and persist in an environment.	[5]
	the interaction of biological, social and environmental determinants that affect a wild animal population's ability to cope with change (stability), recover in the face of change (self-renewal) and meet societal goals (ethic).	[14]
	a multi-disciplinary concept and is concerned with multiple stressors that affect wildlife. Wildlife health can be applied to individuals, populations and ecosystems, but its most important defining characteristics are whether a population can respond appropriately to stresses and sustain itself.	[15]
	the physical, behavioural and social well-being of free-ranging animals at an individual, population and wider ecosystem level and their resilience to change.	[16]
human health	health is a state of complete physical, mental and social well-being and not merely the absence of disease or infirmity.	[17]
animal health	a state of physical and psychological well-being and of productivity, including reproduction.	Studdert *et al.* (2020), as cited in [18]

A similar plurality of definitions characterizes the literature on wildlife health (table 1), but two features stand out. First, wildlife health definitions less consistently adopt a holistic well-being approach, with psychological dimensions of well-being rarely made explicit. Second, while wildlife health definitions are often applicable to individuals, many explicitly extend the concept to populations and, in some cases, prioritize population-level sustainability and resilience. This framing parallels concepts such as population health in humans and herd health in domestic animals, but wildlife health definitions are distinctive in that they frequently integrate individual- and population-level perspectives within a single framework. The concept of wildlife population health has been proposed to formalize this population-focused articulation [5]. The emphasis on the population scale has important management implications, because negative impacts on the health of free-living individuals (whether physical or psychological) may not automatically trigger human intervention. For example, in many scenarios, wildlife managers may regard disease-induced mortality as a normal, and even desirable, mechanism of population regulation, provided that populations remain sustainable [15], and pathogens are an integral part of biodiversity and ecosystem function [19].

This plurality of definitions is not new in debates on the philosophy of health [12]. In line with the normativist school of thought, wildlife health can be understood as a socially constructed concept, shaped by social, cultural and political norms and values rather than reflecting a single objective state of wildlife [5]. Empirical evidence supports this view by showing that wildlife health is understood in diverse ways by different stakeholders, including within the same stakeholder groups [15,20]. This plurality has non-trivial implications for decision-making in wildlife management and policy, as multiple meanings must be recognized and navigated to achieve shared objectives, for example, through the explicit operationalization of agreed wildlife health definitions [15].

Wildlife health as a concept thus asks what it means for wildlife to be healthy. However, the term wildlife health is also widely used as a label for a field of research and practice, for example, in academic programmes (e.g. Master of Science (MSc) in Wild Animal Health), professional roles (e.g. Research Fellow in Wildlife Health) or institutional settings (e.g. National Wildlife Health Center). In this sense, wildlife health answers a different question: what do people who work in this area study or manage? In our experience as researchers, educators and collaborators across wildlife health contexts, this practical usage often treats wildlife health as synonymous with wildlife disease, frequently with an implicit emphasis on infectious disease. These two usages of the term are often conflated, which may cause the concept to become aligned with dominant research foci, such as infectious disease, as training programmes, funding calls and job descriptions may reinforce disease-centric framings that are subsequently internalized to define wildlife health.

**Figure 1.**
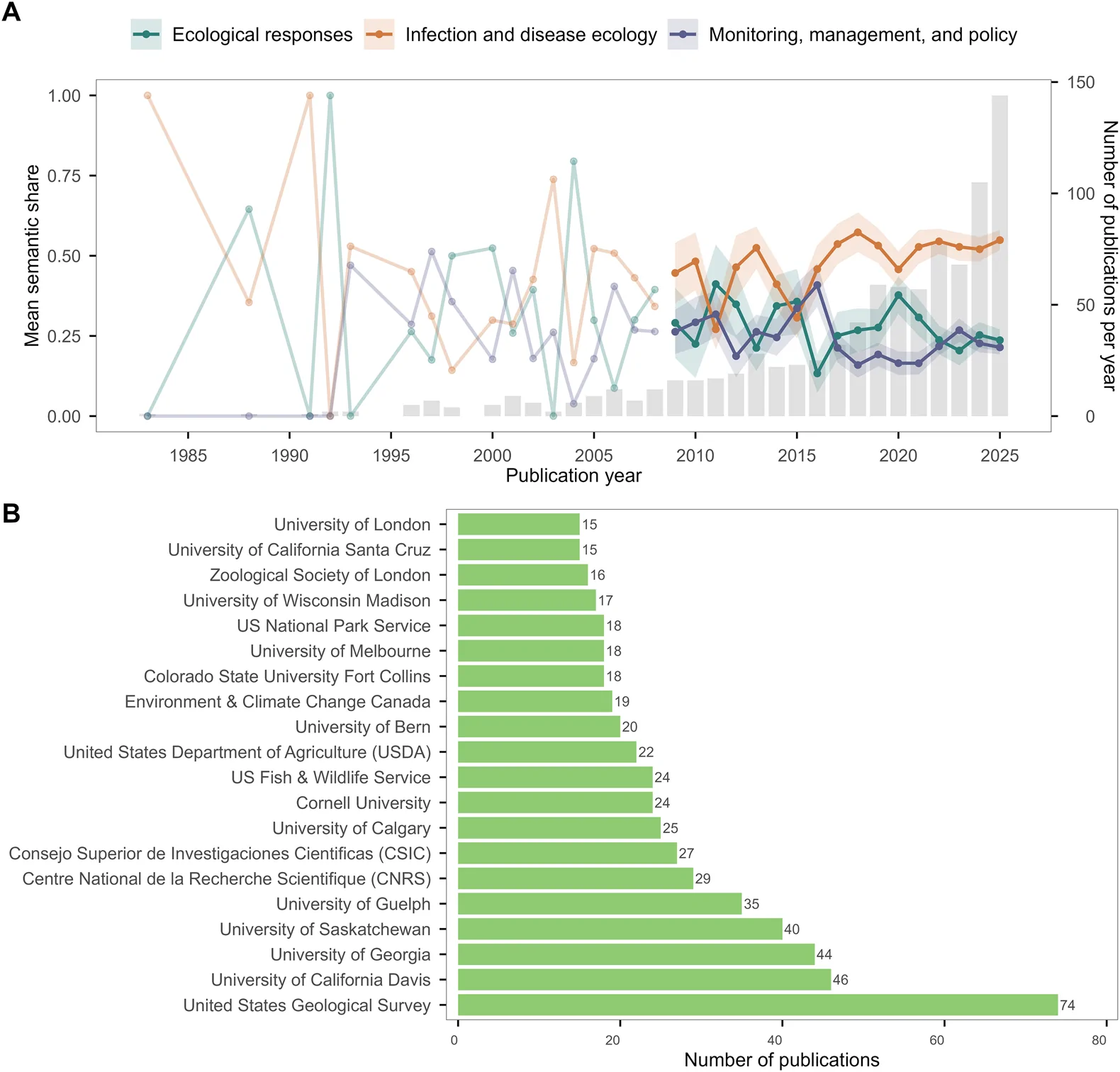
Temporal patterns in conceptual domains and institutional contributions in publications that explicitly include the term ‘wildlife health’. The analysis is based on 902 publications indexed in the Web of Science Core Collection. These analyses characterize patterns of term usage irrespective of whether wildlife health is used as a conceptual framework or simply as a label. (A) Using GloVe-based distributional semantic embeddings, each document was represented as a term frequency–inverse document frequency (TF–IDF)–weighted semantic vector and compared with conceptual-domain centroids derived from clustered vocabulary. Lines show the annual mean within-document semantic share of each domain (i.e. its relative contribution to the semantic content of papers published in a given year); shaded ribbons indicate ±1 standard error. Lines are faded in years with low publication volume (<15 documents). Grey bars show the total number of publications per year, scaled to the right-hand axis. (B) Top institutional affiliations in the corpus. Affiliations were extracted from Web of Science and counted once per publication. Hierarchical parent organizations (e.g. government departments and university systems) were removed when a specific operational unit or campus-level affiliation was also listed on the same publication to avoid inflation from indexing artefacts. Full methodological details are provided in the supplementary material.

The meaning of wildlife health as it is specifically invoked in scientific discourse can also be examined through semantic analysis. To this end, we applied natural language processing methods to a corpus of scientific publications indexed in the Web of Science Core Collection, comprising the titles, abstracts and keywords of articles that explicitly used the term wildlife health (902 publications published between 1983 and 2025; figure 1A). For clarity, when we write ‘the term wildlife health’, we refer to the phrase itself, rather than to wildlife health as a concept or label. Our analysis therefore captures patterns of term usage irrespective of whether the term wildlife health is used as a conceptual framework or as a label. Our objective with this semantic analysis is not to capture all publications relevant to wildlife health, but to examine the contexts in which authors choose to use the term in prominent parts of their publications (i.e. titles, abstracts or keywords). Using distributional semantics, we mapped the semantic structure of this corpus by training GloVe and word2vec word-embedding models (100 dimensions each) [21,22]. By examining how key terms cluster within this semantic space, we identified recurrent groups of semantically related concepts (hereafter, conceptual domains) that characterize the dominant contexts in which the term wildlife health is invoked in the scientific literature. Full methodological details and extended results are provided in the supplementary material.

Across both word-embedding models, we consistently identified three conceptual domains (figure 1A):

— *Infection and disease ecology*: language centred on infectious processes, host–pathogen associations and disease impacts in wildlife, with particular emphasis on zoonotic risk and cross-species disease contexts.
— *Monitoring, management and policy*: institutional and governance-oriented framings of wildlife health, encompassing monitoring activities, organizational capacity and the coordination and implementation of research, management and policy responses.
— *Ecological responses*: organismal, physiological and behavioural responses to environmental stressors and disturbance, including changes in fitness, reproductive traits and immune or physiological function that link environmental pressures to population- and ecosystem-level outcomes.

Analyses of terminology use through time showed that the language associated with *Infection and disease ecology* and *Monitoring, management and policy* remained highly stable, with vocabularies that stayed closely aligned with their respective domain-average vocabularies (supplementary material, figure S5). By contrast, *Ecological responses* consistently used a broader and more heterogeneous vocabulary, but this linguistic profile was also stable through time (supplementary material, figure S5), indicating persistent diversity in terminology rather than progressive conceptual change.

We also quantified domain semantic share using document-level semantic similarity to domain centroids in the GloVe embedding space (normalized within each document so shares sum to one across domains). *Infection and disease ecology* dominated the literature, accounting on average for 50% of the semantic content across documents, followed by *Ecological responses* (27%) and *Monitoring, management and policy* (23%) (supplementary material, table S2; figure 1A). Temporal analyses showed no strong directional change in the relative contribution of these domains within papers from 2009—the earliest year with sufficient publication volume for robust estimation—through 2025 (figure 1A), indicating little evidence for increasing cross-domain integration or shifts in domain prominence.

Overall, these results indicate that the scientific literature explicitly invoking the term wildlife health is framed predominantly through ecological and epidemiological perspectives, with a strong disease-centric emphasis. By contrast, explicit consideration of psychological dimensions of well-being, as well as ethical reflection, remains extremely limited, consistent with the marginal treatment of these aspects in prevailing definitions of wildlife health. This gap highlights the opportunity for more integrative frameworks linking ecology, veterinary, welfare and social sciences with ethical and philosophical perspectives.

## 3. The emergence of wildlife health as a field of practice

Our analysis of the literature reveals that the use of wildlife health as a label predates its explicit conceptual development. Although the importance of wildlife health had been identified by scientists, including Aldo Leopold and Charles Elton, for around a century, this recognition was initially structured around wildlife disease rather than wildlife health as a broader concept [15,23]. The founding of the Wildlife Disease Association in 1951 marked an early formalization of this field.

The explicit use of the term wildlife health is far more recent. We trace its earliest usage to the early 1970s, including its appearance in the name of the U.S. Geological Survey (USGS)'s National Wildlife Health Center founded in 1975, as well as in an organization named the National Wildlife Health Foundation, which was operating in California by at least 1970 as a veterinarian-led group concerned with wildlife health [24]. The National Wildlife Health Center represents one of the earliest sustained institutional articulations of wildlife health as a distinct field of research and practice. It was established by the U.S. Fish and Wildlife Service in response to the 1973 Lake Andes duck plague outbreak, which killed over 40 000 waterfowl and drew weeks of national media attention [25].

By contrast, the earliest use of wildlife health as a concept rather than merely a label in the Web of Science literature appeared in a 1991 article titled *New focus on wildlife health*. In this article, Cohn [25] discusses a range of infectious and non-infectious causes of mass mortality and reproductive failure in wildlife and their relevance for conservation and management. Notably, Cohn was a journalist specializing in zoo and conservation issues rather than a research scientist, suggesting that the concept of wildlife health has historically bridged scientific, institutional and public domains.

Using the Web of Science–indexed publication counts (figure 1A), we examined how use of the term wildlife health has changed over time using segmented regression analyses on both the original count scale and the log-transformed scale, with data up to 2025. Publication frequency increased exponentially after 1985, with the log-linear model explaining 91.3% of the temporal variation. The estimated post-1985 slope was 0.122 (95% CI: 0.103–0.134), corresponding to an average annual increase of approximately 12.9% in the number of publications using the term. On the original count scale, segmented regression identified a breakpoint between 2016 and 2017, indicating a marked acceleration in absolute publication output. After 2016, publication counts increased by an average of 11.2 papers per year (95% CI: 9.2–13.2), compared with 0.75 papers per year prior to this breakpoint. Together, these results indicate that although the term wildlife health appeared sporadically in the Web of Science literature from the early 1980s onward (and, until 1991, only in reference to the National Wildlife Health Center), its widespread adoption is recent, with a rapid acceleration after 2016.

Beyond the marked increase in use of the term, there is additional evidence that wildlife health has developed not only as an area of academic research but also as a field of practice. This is reflected in the establishment of dedicated institutions and collaborative networks (e.g. the Canadian Wildlife Health Cooperative, established in 1992; the UC Davis Karen C. Drayer Wildlife Health Center, established in 1998; Wildlife Health Australia, established in 2002), as well as in the emergence of professional self-reflection, with researchers and practitioners explicitly discussing the scope, structure and governance of wildlife health (e.g. [5,6,15,26,27]). From a governance perspective, an important milestone was the launch of the Wildlife Health Framework in 2020, developed by the WOAH with the goal of protecting wildlife health worldwide to achieve One Health [28].

## 4. Wildlife health within an institutional landscape: the Zoological Society of London as a case study

An analysis of institutional affiliation data from Web of Science–indexed publications that explicitly use the term wildlife health highlights ZSL's prominent position within wildlife health research. ZSL ranks among the top 2% of the most prolific institutions worldwide (18th of 982; figure 1B) and is notably the only conservation NGO among these top-ranked organizations, which are otherwise dominated by universities and public-sector research institutions (figure 1B). An analysis of ZSL's scientific publications indicates a structured and substantial wildlife health research agenda, accounting for 24.8% of the institution's total scientific output and 23.4% of citations (supplementary material).

ZSL's contributions to wildlife health research long predate the formal use of the term. During the institution's first century (from its foundation in 1826 to the early twentieth century), research relevant to wildlife health transitioned from taxonomic description to comparative pathology, with veterinary medicine and pathological studies at ZSL's London Zoo yielding early insights into diseases such as rickets, tuberculosis and parasitic infections [10,11,29]. Following the First World War, ZSL improved its scientific infrastructure: by 1928, the institution had a full-time pathologist, an anatomical fellow and several honorary parasitologists and bacteriologists [10]. This professionalization laid the groundwork for a research-intensive model formalized in the 1960s with the establishment of ZSL's Wellcome Institute of Comparative Physiology and Nuffield Institute of Comparative Medicine [10,11], later combined with ZSL's veterinary group to form the Institute of Zoology (IoZ) in 1976 [11].

Early wildlife health work at the IoZ focused primarily on zoo animals, including notable contributions to understanding the bovine spongiform encephalopathy epidemic in non-domesticated bovids and felids [30]. Research conducted particularly in the 1970s and 1980s also focused on the development of new veterinary anaesthetics, contributing to the wider use of agents such as etorphine and to the establishment of safe and effective immobilization techniques for wildlife, especially large mammals [31]. ZSL also played a foundational role in the development of comparative haematology, drawing on blood samples from the wide diversity of species housed in ZSL's zoos; this work contributed to several influential publications in the field, including *A Colour Atlas of Comparative Veterinary Haematology* [32].

From the 1980s onwards, an increasing proportion of research has been conducted on free-living wildlife in the UK and overseas, with an emphasis on identifying, understanding and mitigating disease threats to conservation. ZSL's leadership in amphibian health research is particularly notable. Since the 1990s, ZSL has contributed to the first identification of a ranavirosis epidemic in free-living amphibians in Europe [33,34] and led a multi-national, multi-disciplinary team that identified the chytrid fungus *Batrachochytrium dendrobatidis* as a major driver of global amphibian declines and extinctions [35]. Together, these contributions helped establish a large and growing body of research on amphibian diseases, their impacts and their mitigation (e.g. [36–40]).

In the early 2000s, the zoo veterinary team was transferred to the auspices of the zoos and became Wildlife Health Services (WHS). Staff from the former veterinary science group whose work focused on free-living wildlife were retained within the IoZ, including researchers working on British wildlife health, South Asian vulture declines and disease risk analysis and health surveillance associated with wildlife translocations. Since then, wildlife health research at the IoZ has been conducted not only by veterinary scientists, but increasingly by researchers whose primary expertise lies in behavioural, evolutionary or population ecology. In parallel, WHS-led veterinary medicine and pathological investigations, together with the work of the ZSL's Evidence-based Animal Care team on welfare, nutrition, behaviour and zoo research, have continued to generate influential research outputs, maintaining a link between animal health, welfare, pathology, research and conservation practice across ZSL's work.

Looking across this history, ZSL's wildlife health research agenda has not been an exception to the wider disease-centred pattern identified in the scientific literature. Rather, it provides an institutional example of the same tendency: wildlife health research has often been organized around the detection, investigation and mitigation of disease threats. This emphasis is also evident in the research topics identified from ZSL publications using embedding-based topic analysis (see supplementary material, table S3). However, disease-centred does not mean restricted to infectious disease. ZSL's work has also addressed non-infectious causes of morbidity and mortality, including toxicosis, bycatch, drowning, neoplasia and nutritional disease, as well as underlying drivers of health such as contaminant exposure and its effects on reproductive function, immune status and population viability (supplementary material, table S3). ZSL research has also increasingly addressed welfare questions. This is most visible in work on wild animals under human care (supplementary material,table S3), but this welfare focus also extends to free-living wildlife, including recent work on health and welfare responses to urbanization and parasitism in grey squirrels (*Sciurus carolinensis*). This history therefore illustrates both the persistence of disease-centred framings in wildlife health and the broader individual- and population-level scope that can emerge from long-term wildlife health research.

### (a) Embedding wildlife health research in policy and practice

A persistent challenge for wildlife health is that, although governments typically hold the legal mandate to manage wildlife, the institutional systems through which health risks are governed have been designed primarily around livestock production, trade and food security rather than the health of free-living wildlife populations. Consequently, wildlife health is frequently addressed using regulatory frameworks, surveillance architectures and professional competencies developed for domesticated animals, with limited capacity to accommodate the ecological complexity, transboundary dynamics and conservation objectives inherent to wildlife systems [26]. This institutional mismatch constrains the ability of government agencies to anticipate, detect and respond to wildlife health risks in ways that are ecologically appropriate and conservation relevant. Partnerships with non-governmental actors, including research institutions, therefore offer a practical means of alleviating these constraints. For example, ZSL's Disease Risk Analysis and Health Surveillance programme has worked in partnership with government bodies in England to assess and manage disease risks associated with wildlife conservation interventions, using structured risk analysis and targeted health surveillance to support decision-making within existing regulatory systems. Recently, ZSL used a structured decision-making approach to support the development of the *England Red Squirrel Recovery Strategy*, commissioned by Natural England, the UK Government's statutory nature conservation body for England. The strategy integrates disease risk from squirrelpox with wider determinants of population health, including competition with grey squirrels, habitat availability, demographic and genetic risks and welfare. It does this while also accounting for public acceptability, cost and socioeconomic benefits in conservation decision-making for national species recovery [41]. These examples illustrate the broader role that organizations conducting research can play in connecting wildlife health evidence with decision-making.

**Figure 2.**
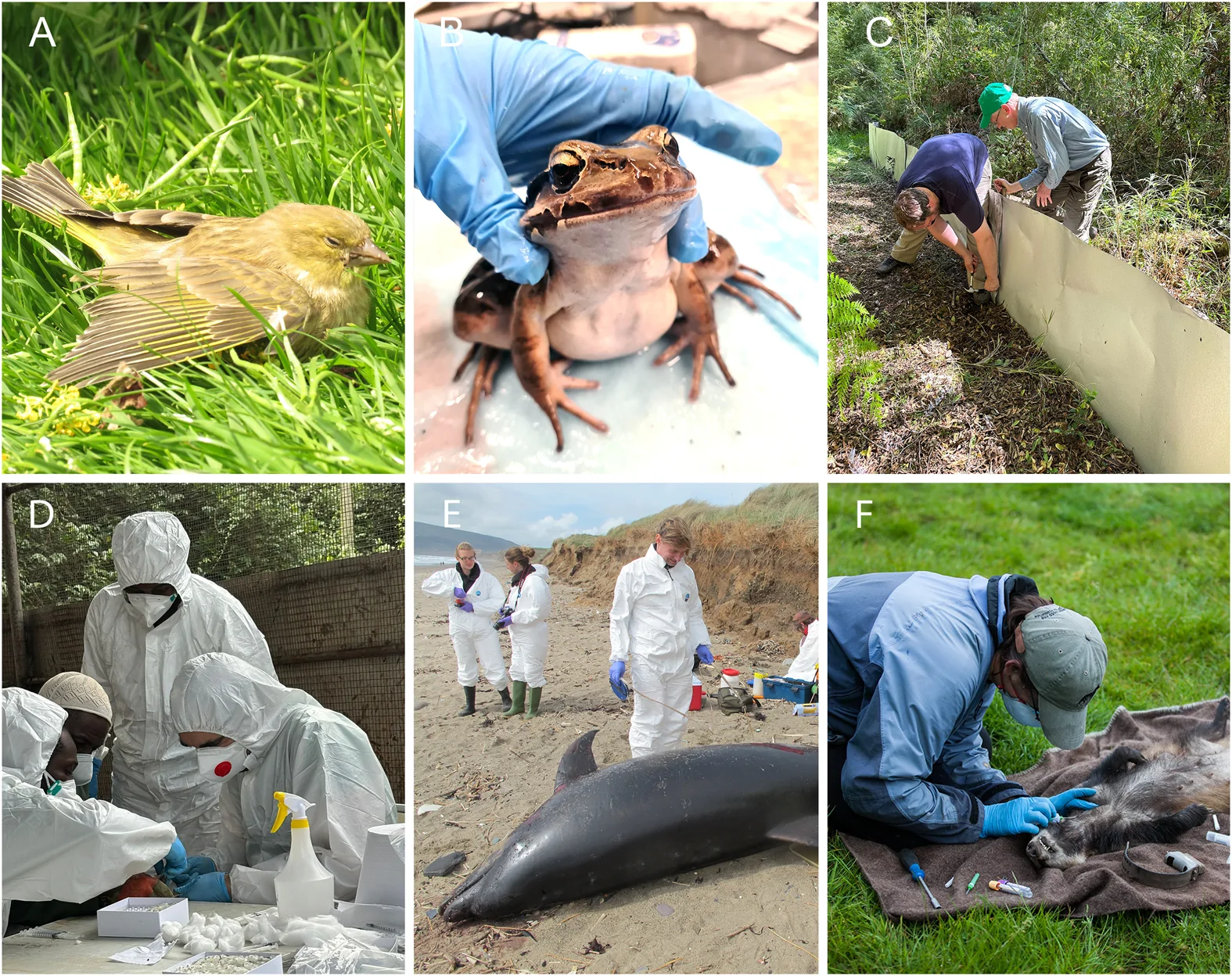
Illustrative examples of ZSL wildlife health case studies. (A) Greenfinch (*Chloris chloris*) reported to the Garden Wildlife Health Project, showing signs of finch trichomonosis, subsequently confirmed on post-mortem examination. This case forms part of a GB-wide citizen-science surveillance programme investigating disease threats to garden wildlife. Credits: Alison Ritchie. (B) Wildlife Health Services (WHS) staff conducting a routine annual health check on an adult mountain chicken frog (*Leptodactylus fallax*) held at London Zoo as part of a multi-institution conservation programme aimed at recovering this critically endangered species endemic to the eastern Caribbean. Credits: ZSL. (C) Exclusion experiment designed to actively prevent co-occurring amphibian species from interacting with Darwin's frog (*Rhinoderma darwinii*). This experiment forms part of a long-term research and conservation project and tests whether exclusion could be an effective mitigation strategy against the chytrid fungus in this fully terrestrial amphibian. Credits: ONG Ranita de Darwin. (D) Blood sampling of an African straw-coloured fruit bat (*Eidolon helvum*) conducted as part of the Bats and Bugs Project, a long-term interdisciplinary research initiative investigating zoonotic virus ecology, immunity and spillover risk in highly mobile bat populations in West Africa. Credits: ZSL. (E) Post-mortem examination of a stranded cetacean conducted under the Cetacean Strandings Investigation Programme (CSIP), a long-term UK government-funded surveillance programme investigating causes of mortality and health threats in stranded marine mammals along the English and Welsh coast. Credits: ZSL. (F) Badger (*Meles meles*) sampled as part of the Badger Vaccination Project in Cornwall, where ZSL works with local landowners, farmers and wildlife groups to develop approaches to control the spread of bovine tuberculosis. Credits: ZSL.

Against this background, we use six representative case studies (figure 2) to examine how ZSL has embedded wildlife health research in policy and practice. Summaries of these case studies follow, with full descriptions provided in the supplementary material.

Garden Wildlife Health provides a participatory model for wildlife health surveillance in species living close to people. Building on ZSL's long-term investigations of garden bird and amphibian mortality, it integrates public reports of sick and dead wildlife with diagnostic investigation, systematic monitoring delivered with conservation partners and multi-sectoral engagement. This approach has enabled the detection of emerging threats, appraisal of public-health risks and quantification of disease-mediated population declines, including greenfinch (*Chloris chloris*) population declines owing to trichomonosis [42–45]. Its translational value lies in connecting wildlife health research with participatory science and contributing to the government-led GB Wildlife Health Partnership, through which key surveillance findings are escalated to policy teams to inform risk assessments and disease management plans.

In WHS, clinical care, pathology and preventive health management for animals under ZSL's care form a pathway through which veterinary expertise contributes to conservation practice. Its work integrates veterinary medicine, diagnostics, molecular tools, tissue banking, research and training, generating evidence that supports animal health in a holistic sense, *ex situ* conservation breeding and threatened-species management. This includes applied health-screening and husbandry approaches for threatened species, such as mountain chicken frogs (*Leptodactylus fallax*) and *Partula* snails, as well as wider capacity-building for wildlife health professionals [46–48]. WHS therefore shows how clinical and pathological expertise developed within zoological institutions can inform conservation beyond those institutions.

The Darwin's Frogs Conservation Project shows how wildlife health research can become central to conservation action for Darwin's frogs (*Rhinoderma darwinii* and *Rhinoderma rufum*). Initial population surveys documented severe declines in both species and the likely extinction of *R. rufum*, while subsequent work identified chytridiomycosis as a major threat to both species [49–51]. Research on pathogen genomics, transmission dynamics, host population ecology and disease impacts has been linked to biosecurity, surveillance, emergency response, *ex situ* assurance colonies and *in situ* disease mitigation trials [50–53]. Through the Binational (Chile-Argentina) Conservation Strategy for Darwin's Frogs, this work connects disease ecology with broader recovery planning, including coordinated *in situ* and *ex situ* conservation action across institutions and countries [54].

Bats and Bugs addresses a human–wildlife interface shaped by zoonotic risk, land-use change and conservation concern. The project combines virology, ecology, genetics and social science to investigate pathogen circulation in African fruit bats, the bat bushmeat trade, human behaviours relevant to zoonotic pathogen spillover and seasonal patterns of viral shedding [55–58]. Long-term partnerships with Ghanaian institutions and government partners have been central to this work [59,60]. Its translational contribution lies in aligning public-health planning with bat conservation and safer coexistence within a One Health framework, rather than treating bats simply as sources of risk.

The Cetacean Strandings Investigation Programme (CSIP) demonstrates the value of long-term national surveillance. Established as a government-funded programme following public and governmental concern about marine mammal mortality, CSIP combines long-term strandings data, systematic post-mortem examinations and extensive tissue archives to investigate causes of morbidity and mortality in vulnerable marine species. Over several decades, it has generated evidence on bycatch [61], contaminant exposure [62], noise-associated mass strandings [63], climate change-driven changes in distribution [64] and other anthropogenic threats. Its research supports national and international reporting frameworks and has informed policy, including UK and EU restrictions on brominated flame retardants, demonstrating how sustained surveillance can make wildlife health evidence policy-relevant.

ZSL's bovine tuberculosis work exemplifies translation in the contested management of European badger (*Meles meles*) culling to control bovine tuberculosis in the UK. Research associated with the Randomised Badger Culling Trial and subsequent field studies showed that culling had mixed and sometimes counterproductive epidemiological effects [65–67]. These findings, together with the social and political controversy surrounding badger culling, helped motivate later work on non-lethal alternatives, including farm husbandry measures and badger vaccination, involving engagement with government, farming stakeholders and local communities [68–70], and culminating in a co-designed strategy for England which shifted the focus to cattle management supplemented by badger vaccination and ended the badger cull [71]. This case shows how wildlife health research can inform policy debates while also developing management options where disease control intersects with agriculture, conservation, animal welfare, public opinion and rural livelihoods.

Taken together, these case studies show that translational wildlife health research cannot be reduced to a single model. The context–process–results framework for translational research [72] provides a useful structure to summarize this. First, the context of this research has been shaped by ZSL's vision of protecting wildlife and the recognition of wildlife health as a central component of achieving this, as well as by institutional capacity, including multi-disciplinary teams, clinical and diagnostic expertise, surveillance systems, training infrastructure and long-term partnerships. It has also been shaped by external influences, including wildlife mortality events, species declines, zoonotic risk, contested disease-management policy, public concern and government-funded surveillance or conservation needs. Second, the case studies highlight that communication between researchers and end users was a key component of the process, allowing collaborative science to be aligned with decision-making. Results became meaningful when outputs were designed for uptake by the wide variety of end users involved, in formats such as scientific papers and reports, surveillance datasets, disease factsheets, training programmes, risk assessments and strategic planning documents. This helped translate outputs into outcomes, including threat detection, disease reporting, conservation planning, policy reporting, professional capacity-building and practical management options, showing that wildlife health research becomes translational when institutional capacity is connected to the decision systems through which conservation, management and policy action occur.

## 5. Future directions for wildlife health in the One Health era

Our review shows that wildlife health has become established as a field, marked by increased use of the term, growing professional self-reflection and institutional self-recognition and recent progress in governance. Here, we outline three priorities we consider relevant for the future development of the field.

### (a) From evidence generation to impact: strengthening translational pathways

Our ZSL case studies illustrate that institutions conducting wildlife health research can play an important role in supporting the development and implementation of wildlife health solutions across policy, management and conservation practice. This involves translational approaches including early engagement with decision-makers to identify where scientific evidence can realistically inform policy or management, co-production of research that aligns ecological relevance with regulatory or conservation needs and the use of structured tools that translate scientific evidence into actionable options.

Translational approaches do not always require immediate application of all research, but rather clarity about intended audiences, decision contexts, timescales of impact and the assumptions under which evidence may be used [72]. Institutions can support this by recognizing translational outputs alongside academic publications and by developing systems to monitor uptake and influence policy and management. Because translational work requires additional time and relational investment, often with limited short-term publication returns, reliance on traditional academic metrics can weaken incentives to engage in translation, particularly for early-career researchers [73]. Institutions seeking translational impact in wildlife health may benefit from explicitly addressing these challenges.

### (b) Beyond disease: integrating psychological well-being into wildlife health

Our exploration of wildlife health highlights limited engagement with holistic views of health as a state of comprehensive well-being (or welfare, as often used when referring to well-being in animals). While incorporating psychological well-being into wildlife health frameworks is conceptually and practically challenging—particularly for free-living wildlife and for taxa that have traditionally fallen outside the remit of welfare science and legislation, such as for most invertebrate species—avoiding it altogether risks narrowing wildlife health to what is easiest to measure rather than what may be most relevant to individual animals or society; a concern amplified by growing societal scrutiny of the legitimacy and humaneness of how people manage and intervene in wild animal populations [74].

We therefore argue that explicitly integrating psychological well-being into wildlife health frameworks is timely. Animal welfare provides an important complementary framework for doing so. The WOAH defines animal welfare as ‘the physical and mental state of an animal in relation to the conditions in which it lives and dies’ [75]. This is not to suggest that wildlife health as a field should supersede the established role of welfare science, or that wildlife health should be treated simply as a component of welfare. Rather, the two frameworks should be understood as distinct but interconnected: welfare science makes behavioural, psychological and affective dimensions of well-being explicit, while wildlife health provides a framework for linking these dimensions with physical condition, population sustainability and ecological resilience. Increased interest within welfare science in wild animals [76], and the emergence of wild animal welfare science as a field of study [77], may support this broadening of scope. A more holistic conception of wildlife health may also help counter tendencies to frame wildlife primarily as sources of pathogens within One Health contexts. Importantly, incorporating psychological well-being does not imply abandoning population-level perspectives, but instead offers a way to link individual-level processes with population sustainability and resilience. We also argue that such integration would benefit from avoiding anthropocentric assumptions by grounding welfare assessment in species-specific biology, validated behavioural and physiological indicators—including those through which affective states can be inferred—and the ecological contexts in which free-living animals persist.

As a starting point, welfare considerations could be incorporated in at least two ways. First, by expanding attention to stressors that affect behaviour, physiology and function without resulting in overt physical disease (e.g. by implementing or expanding existing welfare assessment frameworks [78,79]). Second, by fostering dialogue between wildlife health, welfare science and social science to develop assessment approaches that are ecologically grounded yet sensitive to normative concerns. Measuring welfare, particularly affective states, in free-living wildlife remains challenging [80], and further validation or development of indicators will be required.

### (c) Wildlife health within One Health: from risk framing to reciprocity

The future of wildlife health within One Health depends on how wildlife is positioned conceptually. As One Health becomes increasingly embedded in global governance, there is a risk that wildlife continues to be framed primarily as a source of infection risk to humans and domestic animals. While this framing has driven important investments in surveillance and preparedness, it captures only a subset of the values and functions associated with healthy wildlife.

An alternative, more reciprocal, framing recognizes wildlife health as both an outcome and a driver of healthy social–ecological systems. Wildlife is not merely a reservoir to be managed, but an integral component of ecosystem processes that underpin human well-being in diverse and often indirect ways. Recent operational frameworks for wildlife health within One Health, such as that proposed by Goulet *et al.* [6], provide a starting point for articulating this integrative perspective.

In conclusion, wildlife health has matured as a field, but its future relevance will depend on its ability to evolve conceptually and institutionally. Strengthening translational pathways, broadening wildlife health to focus on comprehensive wildlife well-being and repositioning wildlife as a reciprocal partner within One Health are not separate challenges, but mutually reinforcing ones. Addressing them offers a route towards more integrated, ethically grounded and impactful wildlife health science for the decades ahead.

## Supplementary Material



## Data Availability

Code and data required to reproduce the analyses described in this manuscript and the electronic supplementary material are available on Zenodo [81]. Supplementary material is available online [82].
