# Supplementary material for: Wildlife health in the One Health era: lessons from a 200-year institutional case study
Source: Proc Biol Sci. 2026 Sep 16;293(2079):20260285. doi: 10.1098/rspb.2026.0285 (PMC13577807; doi:10.1098/rspb.2026.0285)
Supplement: Supplementary file 1 [file rspb.2026.0285.zip › rspb.2026.0285/rspb20260285_si_001.pdf]

Electronic Supplementary Material

Wildlife health in the One Health era: lessons from a 200-year institutional case study

Andrés Valenzuela-Sánchez, Javier Sánchez-Romano, Becki Lawson, Katharina Seilern-Macpherson, Rob Deaville, Rosie S. Williams, Rosie Woodroffe, Louise Gibson, Amanda Guthrie, Simon Spiro, María Díez-León, Stuart Patterson, Benjamin Tapley, Sarah Haddow and Andrew A. Cunningham

Journal: Proceedings of the Royal Society B: Biological Sciences
Article DOI: 10.1098/rspb.2026.0285

Contents

Semantic analysis of publications using the term "wildlife health" 2
Literature search and corpus construction 2
Word-embedding models 3
Local Semantic Neighborhoods and clustering 4
Interpreting clusters: conceptual domains 6
Cross-model comparison of domain structure 8
Semantic share of domains in the literature and temporal trends 9
Bibliometric analysis of ZSL publications 13
Embedding wildlife health research in policy and practice: ZSL case studies 17
Garden Wildlife Health: participatory wildlife health surveillance 17
Wildlife Health Services: modern-day veterinary care of captive wildlife at ZSL 18
Darwin's Frogs Conservation Project: species conservation in Chile and Argentina 19
Bats and Bugs Project: interdisciplinary research in emerging pathogens in West Africa 21
Cetacean Strandings Investigation Programme: long-term national wildlife health surveillance 22
Bovine tuberculosis management in the United Kingdom 23
Code 25
References 26
Tables 33
Figures 46

Semantic analysis of publications using the term “wildlife health”

In this section, we present our semantic characterisation of the discourse represented by publications that explicitly use the term “wildlife health.” We use natural language processing (NLP) techniques to examine how researchers conceptualise this concept.

Literature search and corpus construction

We constructed a corpus for systematic analysis by searching the term “wildlife health” in the topic, title, abstract, author keywords, and Keywords Plus fields of the Web of Science Core Collection. The search was conducted on 25 December 2025 and returned 940 records, with the earliest publication dating to 1983. After removing records lacking abstracts, 902 documents were retained for analysis.

To assess the coverage of Web of Science, we conducted an equivalent search in PubMed on 16 January 2026. This search returned 690 records, of which 35 were not present in the Web of Science dataset. Closer inspection revealed that 13 of these records had been added to PubMed in December 2025 or January 2026, indicating that they were likely unavailable in Web of Science at the time of the original search. The remaining PubMed-only records comprised preprints, book chapters, articles from journals with limited or inconsistent indexing, and a few very recent publications likely subject to indexing lag. After accounting for these factors, the combined database comprised 962 records, of which 97.7% were indexed in Web of Science. This demonstrates the high coverage of Web of Science for the wildlife health literature within the scope of this study and supports its use as the sole source database for the analyses presented here.

We merged titles, abstracts, Author Keywords, and Keywords Plus into a single text field and processed it using standard NLP steps (HTML-entity decoding, lowercasing, punctuation removal, and whitespace normalisation). To preserve

semantically meaningful compounds, selected hyphenated expressions (e.g., well-being, self-renewal) were normalised to single tokens prior to punctuation removal.

Tokenisation of the cleaned corpus produced 16,601 unique unigrams, which we refined to 2,944 terms by removing stopwords, excluding tokens shorter than three characters, and applying frequency and document-proportion thresholds (at least 10 occurrences; appearing in at least 0.1% of documents). This pruning reduces noise and stabilises semantic modelling. The resulting document–term matrix (902 × 2,944) was, as expected for specialised scientific literature, highly sparse (i.e., each article uses only a small fraction of the overall vocabulary), but retained adequate lexical density (approximately 94 non-zero terms per document on average), enabling stable embedding training and the recovery of interpretable conceptual structure.

Word-embedding models

The curated corpus served as the basis for two complementary word-embedding models. Word-embedding methods learn how words in a corpus relate to one another from their patterns of use. In these models, each term is encoded as a dense vector in a continuous, low-dimensional space, so that words used in similar contexts tend to have similar representations [1,2]. Specifically, we produced: (i) a 100-dimensional GloVe model [1], which represents global co-occurrence structure; and (ii) a 100-dimensional word2vec skip-gram model [2], which represents local predictive context. Both models were trained using a context window of five words. Together, these models provide distinct yet complementary perspectives on how wildlife health terminology is organised.

GloVe is explicitly built from global statistics: it factorises a large word–word co-occurrence matrix so that the dot product between two word vectors approximates a transformed co-occurrence score (e.g., log counts or pointwise mutual information) [1]. In practice, this means that each GloVe vector integrates information from the entire corpus, smoothing over local idiosyncrasies and emphasising broad regularities in how terms co-occur across documents. As a result, GloVe tends to capture relatively stable, corpus-level associations (e.g., links between “zoonotic”,

“spillover” and “reservoir”) and can be interpreted as a compact, low-rank summary of the global co-occurrence structure [3,4].

By contrast, word2vec skip-gram is a local, predictive model: it learns embeddings by training a shallow neural network to predict words that occur within a fixed window around a target term [2]. Rather than directly factorising a global matrix, the model updates vectors based on many local context windows, using techniques such as negative sampling to distinguish observed word–context pairs from random noise. This makes word2vec particularly sensitive to the immediate lexical environments in which a term appears, often capturing fine-grained syntactic and topical patterns.

Taken together, these properties imply that agreement between GloVe and word2vec embeddings provides relatively robust evidence for a conceptual association grounded in both global and local usage, whereas discrepancies between the two may highlight cases where terms share broad corpus-level co-occurrence but appear in systematically different local contexts, or vice versa [3,4].

Local Semantic Neighborhoods and clustering

To evaluate how wildlife health discourse represents different facets of the “health” concept, we defined a set of 53 seed terms (table 1) that synthesise definitions of health in wildlife, animal, and human contexts (as outlined in table 1 of the main text) together with additional relevant terms identified by us. Of these, 39 were retained after filtering for terms that appeared at least 10 times or in at least 0.1% of the documents, thereby removing terms that are seldom used in our corpus (table 1). For each of the 39 retained seed terms, we identified its 15 nearest neighbours using cosine similarity, yielding 585 seed–neighbour pairs in both the GloVe and word2vec models.

We then generated two-dimensional conceptual maps by applying principal component analysis (PCA) [5] to the embeddings of seed terms and their neighbours. In the GloVe map (figure S1), PC1 and PC2 explained 5.6% and 3.1% of the variance, respectively; in the word2vec map (figure S2), they explained 5.2% and 4.9%. These proportions are typical of PCA applied to high-dimensional

embeddings and indicate that such maps provide exploratory visualisations capturing broad semantic gradients rather than fine-grained structure.

Despite the limited variance captured by the PCA, a coherent pattern emerges across both embedding models. The seed terms retained after frequency filtering indicate that wildlife health discourse is dominated by ecological, physiological, and epidemiological terminology. Seed terms explicitly associated with mental or psychological dimensions of health were excluded due to low frequency or lack of representation in the embedding space. The terms *wellbeing* and *welfare* were retained but did not form a distinct conceptual cluster; instead, they appeared embedded within regions dominated by monitoring, management, and policy-related concepts.

In the word2vec semantic space, *welfare* is most strongly associated with terms such as *animal*, *handling*, *care*, *captivity*, and related management practices (figure S2). These associations are based on cosine-similarity nearest neighbours identified in the original 100-dimensional embedding space, indicating that welfare-related concepts in this corpus are framed primarily in terms of animal management and conservation practice. By contrast, *wellbeing* is associated with more institutional and system-oriented terms, including *human*, *sanitary*, *habitats*, and *domains*, suggesting a governance- and policy-oriented framing rather than a focus on mental or subjective dimensions. In the GloVe model, similar distinctions are evident, with *welfare* clustering around animal- and environment-oriented terms, while *wellbeing* appears in more abstract, evaluative, and institutional contexts.

Using the unique terms appearing in the seed–neighbour pairs (GloVe: 432; word2vec: 446), we applied k-means clustering using R's `kmeans` function to identify broader conceptual domains in the wildlife health corpus. We evaluated three- and four-cluster solutions; while the three-cluster configuration yielded clear, coherent conceptual domains, the four-cluster alternative introduced an additional diffuse "catch-all" group and did not improve interpretability, so we retained the three-domain structure for subsequent analyses.

Cluster sizes ranged from 63 to 256 terms in the GloVe model and from 126 to 177 terms in the word2vec model. For each cluster in each model, we identified the ten

most representative terms—those with highest cosine similarity to the cluster centroid—which formed the basis for interpreting the resulting conceptual domains.

Interpreting clusters: conceptual domains

Cluster labels were assigned manually based on our interpretation of the top representative term sets (table 2), taking into account consistent semantic themes across the GloVe and word2vec representations. This procedure yielded the following domain labels:

Infection and disease ecology: This domain captures the core language of wildlife disease ecology and pathogen transmission, and it is the most semantically aligned across both embedding models. In the GloVe model, representative terms (*wildlife*, *disease*, *health*, *diseases*, *human*, *conservation*, *impact*, *zoonotic*, *risk*, *environmental*) reflect a broad co-occurrence structure linking wildlife health to environmental context and cross-species disease risk. In the word2vec model, the same conceptual space is populated by more pathogen- and host-specific terms (*fever*, *francisella*, *ehrlichia*, *hemorrhagic*, *infectious*, *zoonotic*, *swine*, *acari*), consistent with its greater sensitivity to local context and named entities. Together, these patterns indicate a domain organised around infection processes, host–pathogen associations, and zoonotic risk, with word2vec sharpening the domain into more clinically and taxonomically explicit language.

Monitoring, management, and policy: This domain reflects institutional, programmatic, and governance-oriented framing of wildlife health, supported by both embedding models but expressed at different levels of abstraction. In the GloVe model, representative terms (*ecosystem*, *monitoring*, *national*, *center*, *veterinary*, *research*, *capacity*, *integrated*, *ecological*, *medicine*) emphasise the evaluative and organisational infrastructure of wildlife health, including monitoring systems, institutional capacity, and formal research and management contexts, often at national or organisational scales. In the word2vec model, the domain is characterised by coordination- and implementation-oriented governance language (*governance*, *interdisciplinary*, *health*, *sciences*, *wildlife*, *curriculum*, *plans*, *implement*, *initiatives*, *management*), suggesting that wildlife health is framed as an

applied problem requiring organised, cross-sectoral action. The alignment across models supports interpreting this as a monitoring, management, and policy domain that spans technical monitoring and the institutional mechanisms used to design and implement responses. Although the term “surveillance” clusters within the *Infection and disease ecology* domain in both embedding models, it lies at the semantic boundary with this governance-oriented domain, reflecting its dual role as both an epidemiological practice and an institutional response mechanism.

Ecological responses: This domain captures organismal and ecological responses to environmental pressures and disturbance, characterised by terms describing stress, physiological performance, and system-level change. In the word2vec model, representative terms (*stressors*, *physiological*, *immune*, *traits*, *fitness*, *reproductive*, *physiology*, *disturbances*, *behavioural*, *urbanization*) emphasise response pathways and performance consequences under environmental stress, including behavioural and physiological responses that can scale up to demographic and ecological outcomes. In the GloVe model, the domain is anchored by a more heterogeneous vocabulary (*salmonella*, *biotic*, *persistence*, *costs*, *distance*, *iron*, *young*, *shot*, *allows*, *goals*), reflecting the diverse exposure contexts, organismal states, and case-specific settings through which ecological responses are discussed in the literature. Together, these patterns support interpretation of this domain as ecological responses: the language of disturbance, stress, and organismal performance under environmental change.

We visualised each clustering solution by projecting all clustered terms onto the first two principal components of their respective embedding spaces (figures S3 and S4). In both models, the resulting maps revealed distinct but partially overlapping semantic regions, indicating that the three-domain structure captures a broad and interpretable organisation of concepts within the wildlife health literature. However, the two embedding methods differed in how sharply these domains were delineated. In the GloVe map, domain boundaries were comparatively diffuse, with substantial overlap among clusters, consistent with GloVe’s reliance on corpus-wide co-occurrence patterns that emphasise broad thematic associations rather than fine-grained contextual distinctions. In contrast, the word2vec map showed more clearly structured gradients and locally coherent regions, with sharper separation between

domains despite some overlap, reflecting the model's sensitivity to local contextual usage. Together, these visual patterns support the robustness of the three-domain solution while highlighting meaningful model-specific differences in how semantic structure is expressed in reduced-dimensional space.

Cross-model comparison of domain structure

To assess whether conceptual domains were consistently recovered across embedding methods, we compared GloVe and word2vec clusters using the Jaccard similarity coefficient [6], calculated on the top ten representative terms assigned to each domain, alongside expert qualitative interpretation of the full cluster vocabularies, thematic content, representative terms, and definition-seed structures. Expert assessment focused on whether clusters expressed the same underlying conceptual domains, irrespective of the specific lexical items selected by each embedding model.

Quantitative cross-model agreement based on representative-term overlap was low. Across all GloVe–word2vec domain pairings, the mean Jaccard similarity was 0.032, with a maximum value of 0.176. The highest overlap occurred for the *Infection and disease ecology* domain when compared directly across models (Jaccard = 0.176), indicating limited but non-trivial lexical agreement for this core domain. Notably, the same GloVe infection–disease cluster also showed a secondary overlap with the word2vec *Monitoring, management, and policy* domain (Jaccard = 0.111), reflecting shared boundary vocabulary associated with health, risk, and institutional response.

The remaining domains showed weaker correspondence. The *Monitoring, management, and policy* domain displayed no representative-term overlap with its word2vec analogue, consistent with a diffuse and context-dependent lexicon that is realised differently by GloVe and word2vec. The *Ecological responses* domain likewise showed minimal cross-model overlap, indicating that each embedding model selected largely distinct lexical exemplars to represent similar response-oriented ecological concepts.

Despite low Jaccard similarity values, expert qualitative evaluation indicated strong conceptual correspondence between GloVe and word2vec clusters. Both models recovered the same three broad thematic domains—*Infection and disease ecology*, *Monitoring, management, and policy*, and *Ecological responses*—even though they differed substantially in their lexical realisations and boundary placements. These results indicate that representative-term overlap substantially underestimates conceptual similarity, as GloVe and word2vec frequently encode the same underlying constructs using different vocabularies.

Semantic share of domains in the literature and temporal trends

We quantified the within-document semantic share of each of the three conceptual domains in the wildlife–health literature and examined how these shares have changed through time. For this analysis we focused on the GloVe embedding model. Using a single embedding space avoids incompatibilities between models and ensures that document–domain comparisons based on cosine similarity are mathematically meaningful. This choice was guided by explicit evaluation of domain separability in embedding space. In the GloVe model, domain centroids were directionally distinct (pairwise cosine similarities ranging from –0.40 to 0.34), supporting meaningful discrimination among domains in centroid-based document similarity analyses. In contrast, domain centroids in the word2vec space were highly aligned (pairwise cosine similarities ranging from 0.61 to 0.65), indicating a strong shared semantic component across domains. Importantly, this alignment does not imply an absence of domain structure in word2vec; rather, it indicates that domain distinctions in this embedding are encoded primarily through local contextual relationships rather than global directional separation of centroids, which limits the interpretability of centroid-based semantic-share estimates. We therefore base semantic-share and temporal trend analyses on the GloVe embedding space.

For each conceptual domain we calculated a domain centroid as the mean embedding of all terms assigned to that domain cluster, followed by L2 normalisation to ensure comparability across domains. These centroids provide fixed semantic reference points for evaluating the alignment of individual documents within the

embedding space [6]. The most representative terms within each cluster were used for domain interpretation and labelling, whereas centroid construction incorporated the full cluster vocabulary to capture the broader lexical footprint of each domain.

Each of the 902 scientific articles retrieved from the Web of Science search was then represented as a document-level semantic vector using TF–IDF-weighted word embeddings. Term Frequency–Inverse Document Frequency (TF–IDF) assigns higher weight to terms that are frequent within a document but relatively rare across the corpus, thereby emphasising conceptually informative words over ubiquitous but semantically uninformative terms [7,8]. Document embeddings were obtained by multiplying each word embedding by its TF–IDF weight and averaging across all words in the document, an approach consistent with established baselines for embedding-based document representations [9].

Cosine similarity between each document vector and each domain centroid was used to quantify semantic alignment. These similarity values reflect the absolute strength of association between documents and domains, and documents may exhibit non-zero similarity to multiple domains simultaneously. To estimate the relative prevalence of domains across the literature, domain-specific similarity scores were subsequently normalised within each document to sum to one, yielding a within-document semantic share for each domain. Mean similarity therefore captures the overall strength of alignment with a given domain, whereas semantic share quantifies the relative contribution of each domain within documents, providing a compositional measure of domain emphasis across the literature. These shares were averaged across documents to characterise domain prevalence overall and through time. This continuous, threshold-free approach avoids distortions introduced by arbitrary similarity cut-offs and accommodates both dominant and diffuse expressions of conceptual domains within individual articles.

Infection and disease ecology exhibited the largest mean semantic share across documents (mean cosine similarity = 0.198; mean semantic share = 0.498). *Ecological responses* constituted a substantial secondary domain (mean similarity = 0.0379; mean share = 0.271), while *Monitoring, management, and policy* contributed a smaller but still meaningful proportion of semantic content overall (mean similarity = 0.120; mean share = 0.231). These patterns indicate that the literature is

dominated by infection- and disease-oriented framing, with ecological-response language contributing a substantial share of semantic content and governance-oriented framing contributing a smaller share on average.

We assessed temporal changes in the lexical structure of the three conceptual domains using GloVe-based semantic drift. For each domain, yearly term-frequency–weighted centroids were constructed from domain-associated vocabulary and compared with a corpus-wide baseline centroid. Analyses were restricted to years with at least 15 publications to ensure adequate sampling. Across domains, drift values were largely stable through time, indicating limited directional change in the core terminology associated with each domain despite substantial growth in publication volume (figure S5).

Infection and disease ecology exhibited consistently low drift values (approximately 0.105–0.132) with minimal interannual variation, indicating a highly stable lexical structure. *Monitoring, management, and policy* showed moderately higher but similarly stable drift (approximately 0.320–0.366), suggesting gradual diversification around a persistent conceptual core. In contrast, *Ecological responses* showed substantially higher absolute drift values (approximately 0.523–1.027), indicating that the vocabulary associated with this domain is consistently distinct from the dominant language of the wildlife–health literature. Importantly, drift values for this domain were also stable through time, suggesting a persistent and coherent lexical profile rather than ongoing change or diversification. Overall, the post-2009 record shows no evidence of major temporal shifts in the semantic structure of any domain, with all three exhibiting internally consistent trajectories.

Temporal patterns in domain semantic share were examined by averaging within-document semantic shares by publication year. From approximately 2009 onward—when annual publication volume becomes sufficient for stable estimation—all three domains exhibit broadly stable trajectories through time, with moderate interannual variability but no strong directional trends (figure S6). *Infection and disease ecology* consistently remains the dominant domain, while *Ecological responses* and *Monitoring, management, and policy* maintain relatively constant secondary and tertiary contributions, respectively. Apparent fluctuations in earlier years coincide with very small numbers of publications and are therefore not interpreted

substantively. Overall, these results indicate that although publication volume has increased substantially, the relative distribution of semantic attention across the three conceptual domains within individual papers has remained broadly stable through time, indicating no systematic increase in cross-domain integration in the wildlife–health literature.

Bibliometric analysis of ZSL publications

On 2 December 2025, we searched the Web of Science Core Collection using *Zoological Society of London* as the affiliation term, which returned 5,012 publications, with the oldest record dating to 1916. Web of Science affiliation metadata were used to identify institutional outputs following standard indexing procedures [10,11].

We defined publication eras a priori based on a combination of institutional history and exploratory analysis of annual publication counts. Three breakpoints were used. First, 1926 was selected to separate an initial foundational period characterised by negligible output from a phase of sustained publication activity; this year also corresponds to the earliest structural transition identified in exploratory segmented analyses of the full time series. Second, 1961 marks the establishment of ZSL's research institutes (see main manuscript for details), representing a major institutional shift towards organised scientific research. Third, 2000 was used to delineate the onset of the modern expansion era, corresponding to a visually evident acceleration in publication output and the broader institutional transition to digital, globally networked science. These breakpoints defined four eras: a foundational period (≤1926), an early sustained output period (1927–1960), early research institutes and consolidation era (1961–1999), and the modern expansion era (≥2000).

Using these historically defined eras, we analysed annual ZSL publication counts with negative binomial regression. Publication output differed strongly among eras ($p < 0.001$), increasing by approximately 20-fold following the establishment of the ZSL's research institutes (rate ratio = 20.6, 95% CI: 8.3–50.7) and by more than 80-fold in the post-2000 period (rate ratio = 86.1, 95% CI: 34.9–212.5) relative to the foundational era. Models allowing era-specific growth rates showed little or no growth prior to 1961, statistically significant positive growth during the early research institutes and consolidation era (3.1% per year, 95% CI: 1.8–4.4%), and a substantially steeper growth rate after 2000 (6.8% per year, 95% CI: 5.3–8.3%), consistent with an approximately exponential expansion of research output in the

modern era. Visual inspection suggests that output peaked around 2020, after which growth appeared to plateau (figure S7A).

We performed an embedding-based topic analysis on this corpus. Titles and abstracts were extracted and concatenated into a single text field per record. All publications contained non-empty text fields and were retained for analysis (n = 5,012). Abstract text was available for N = 3,916 publications (78.1%). Text was embedded using OpenAI's *text-embedding-3-large* model, producing 3,072-dimensional semantic vectors [12]. Embeddings were computed in batches to avoid token-limit constraints.

To reduce noise and align the embedding space across documents, we applied principal component analysis (PCA) [5] and retained the first 50 components, which together explained approximately 45.6% of the total variance. We then identified stable semantic topics using a modern density-based workflow following established best practices in machine-learning bibliometrics [13]. Specifically, we projected the PCA-reduced embedding space into a 15-dimensional manifold using Uniform Manifold Approximation and Projection (UMAP) [14], with `n_neighbors = 40` and `min_dist = 0.1`, and applied Hierarchical Density-Based Spatial Clustering of Applications with Noise (HDBSCAN) [15] with `minPts = 20` to identify dense topical clusters. This approach does not require the number of topics to be pre-specified and naturally treats diffuse documents as noise.

The final model identified 47 research topics (table 3), with cluster sizes ranging from 21 to 206 publications (median = 57), and encompassing a total of 3,311 articles (66.1% of the total). In contrast, 1,701 articles (33.9%) were classified as noise, representing publications that did not belong to any dense semantic neighbourhood and were therefore not assigned to any of the 47 topics.

For interpretability, we generated human-readable topic names and descriptions using a large language model. For each cluster, we randomly sampled up to 15 representative publications (titles and abstracts) and prompted GPT-4.1-mini to (i) suggest a concise topic label (six words or fewer), (ii) provide a 2–3 sentence thematic description, and (iii) extract 3–5 representative keywords. Outputs were returned in structured JSON format and manually checked for coherence. This

approach follows emerging best practice for combining unsupervised semantic clustering with large language models to improve topic interpretability [16,17].

We manually classified the 47 topics into two categories: 15 topics were classified as *Wildlife health* (1,244 papers; 24.8% of the total articles, including those classified as noise), and 32 were classified as *Other topics* (2,067 papers; 41.2% of the total articles). *Other topics* included a variety of research areas, such as macroecology and ecosystem ecology, biodiversity monitoring methods and techniques, conservation planning, genetics, and other conservation-related fields.

To explore temporal variation in the magnitude of each high-level category in the ZSL scientific output, we analysed publication counts aggregated by year and by high-level category. These counts were computed directly from publication-year metadata, without smoothing, interpolation, or imputation. This aggregation provides a complete overview of temporal changes in ZSL research output (figure S7A).

To provide an exploratory measure of impact, we analysed citation counts for the ZSL publications (figure S7B). We used Web of Science Core Collection “Times Cited” as a conservative indicator because it is based on a selectively curated and consistently indexed set of journals, books, and conference proceedings, and it generally yields lower citation totals than broader sources (e.g., Google Scholar) that also capture citations from a wider range of document types (including grey literature) and less standardised records. Accordingly, our WoS-based estimates should be interpreted as conservative (lower-bound) citation counts, offering a transparent and reproducible basis for comparing citation distributions across the high-level categories. WoS Core Collection citation counts were available for all articles, yielding a total of 267,663 citations. Citation statistics differed across the three high-level categories (*Wildlife health*, *Other topics*, *Noise*). The *Wildlife health* category accrued 62,648 citations (23.4% of total citations), with a mean citation count of 50.4, a median of 19.5, and an interquartile range (IQR) of 41. The *Other topics* category had the highest total number of citations, with 126,361 citations overall (47.2% of total citations), and also showed the highest mean citation count (61.2), while the median was 19.0 and the IQR was 48, again reflecting considerable dispersion and skewness. The *Noise* category accrued 78,654 citations in total (29.4% of total citations), with a mean of 46.3 citations per paper; the median citation

count was lower than in the other categories (14.0), with an IQR of 37, indicating a strongly right-skewed distribution. Overall, across all categories, mean citation values were substantially higher than medians, underscoring the influence of a small number of highly cited papers.

Embedding wildlife health research in policy and practice: ZSL case studies

Here, we present six wildlife health case studies from ZSL that illustrate how translational approaches have been developed and implemented.

Garden Wildlife Health: participatory wildlife health surveillance

ZSL began investigating causes of garden bird and amphibian mortality incidents in the early 1990s [18,19]. The Garden Wildlife Health project (GWH), launched in 2013, brought together ZSL's citizen science wildlife health surveillance programmes (the Frog Mortality Project, since 1992, and the Garden Bird Health *initiative*, since 2005) and added health surveillance for the Western European hedgehog (*Erinaceus europaeus*) and reptiles. GWH is a multidisciplinary project delivered through a partnership with the British Trust for Ornithology (BTO), Froglife, and the Royal Society for the Protection of Birds, with project staff including veterinarians, microbiologists and biologists.

GWH champions the use of citizen, or participatory, science to deliver national scanning surveillance of species with close human-wildlife interfaces. Members of the public submit opportunistic reports of sick or dead wildlife online via gardenwildlifehealth.org. Systematic surveillance is achieved through the BTO's Garden BirdWatch network, whose participants provide data on wildlife sightings in their garden on a weekly basis, and note whether or not signs of ill health were observed. This helps control for bias due to variation in observer effort. Analyses integrating disease surveillance and long-term population monitoring datasets have enabled identification and quantification of disease-mediated population declines, for example due to finch trichomonosis [20] (figure 2A). GWH surveillance findings also helped evidence the likely absence of *Batrachochytrium salamandrivorans* in wild urodeles in Great Britain [21]. GWH translates peer-reviewed research into freely accessible online disease factsheets and best-practice guidance.

GWH convenes a multisectoral forum to promote collaboration amongst those with an interest in wildlife health across government, animal welfare and conservation NGOs, horticultural organisations, wildlife rescue centres and trade. Surveillance has led to emerging threat detection, for example ophidiomycosis in wild snakes in Europe [22], and Usutu virus as the first mosquito-borne viral zoonosis identified in the UK [23]. Toxicological studies using the GWH tissue archive revealed widespread exposure of hedgehogs to second generation anticoagulant rodenticides with potential health implications to this species of conservation concern and relevance to pesticide stewardship considerations [24]. Collaboration with national public health laboratories has enabled appraisal of public health risks from a range of enteric pathogens infecting British wildlife, including *Salmonella* Enteritidis [25] and *Escherichia albertii* [26].

GWH contributes to the government's GB Wildlife Health Partnership and key surveillance findings are escalated to policy teams to inform risk assessments and disease management plans. For example, greenfinch (*Chloris chloris*) population declines due to trichomonosis led to the species being Red-listed in the UK, and are adversely impacting the government's UK Wild Bird Population index for farmland generalist species [27].

Wildlife Health Services: modern-day veterinary care of captive wildlife at ZSL

The current WHS team at ZSL brings together veterinarians, veterinary nurses, pathologists, technicians, microbiologists and molecular biologists who deliver integrated wildlife medicine across ZSL's zoos. Using advanced tools such as qPCR, digital radiography and CT, the team provides year-round clinical care, preventive health management, disease investigation and postmortem diagnostics to safeguard the welfare of captive animals in the zoos and also contribute to ZSL's biobank of animal tissues.

Alongside this veterinary work, WHS undertakes research, ranging from case series and mortality reviews to discoveries of new conditions. An example is a retrospective review of mortality events in Socorro doves (*Zenaida graysoni*), an extinct-in-the-wild

species with a current global population below 200 animals [28]. WHS research has also focused on the interplay between zoo nutrition and infectious disease, such as synergies between high iron and high cholesterol diets with yersiniosis and angiostrongylosis, respectively, allowing for *in vivo* studies without the use of experimental animals [29,30]. Tissue archives also contribute to larger studies through collaboration with other research institutions, such as anatomical studies on banked brains [31].

WHS plays a major role in training and mentoring veterinary students, postgraduate trainees and conservation professionals worldwide, ensuring that clinical excellence, scientific expertise and education remain interconnected within ZSL's mission. Within wider, cross-departmental ZSL training programmes, WHS has contributed expertise and supervision to the paired MSc in Wild Animal Health and MSc in Wild Animal Biology, the ECZM residency programmes in Zoo Health Management and Wildlife Population Health, all delivered jointly with the Royal Veterinary College, and the field-based course Interventions in Wild Animal Health (delivered with the Wildlife Health Bridge; [32]).

WHS staff have led applied wildlife health research while also supervising a wide range of student research projects, ensuring that the evidence generated not only improves the health and welfare of captive wildlife but also directly informs conservation of threatened species in the wild, including work on the Mountain chicken frog (*Leptodactylus fallax*; figure 2B) [33], Partula snails (*Partula* spp.) [34], and the Western European hedgehog [35]. This integration of clinical practice, pathological investigations, research, teaching, and both *in situ* and *ex situ* conservation action reflects ZSL's commitment to a holistic approach to wildlife health.

Darwin's Frogs Conservation Project: species conservation in Chile and Argentina

This project began in 2009 as PhD research by a ZSL student, initially assessing the population status and threats facing Darwin's frogs (*Rhinoderma rufum* and *R. darwinii*) in Chile and Argentina. Early field surveys revealed the likely extinction of

R. rufum and widespread declines of *R. darwinii* [36], informing subsequent IUCN Red List and Green Status assessments. In parallel, the project identified the amphibian chytrid fungus (*Batrachochytrium dendrobatidis*; Bd) as a major threat, establishing wildlife health as central to conservation efforts [37].

By culturing Bd isolates from the wild, we revealed a relatively recent introduction of the pathogen into Chile, likely during the 1970s [38], and contributed to a landmark global study showing that the Bd Global Panzootic Lineage originated in East Asia in the early 20th century and spread worldwide alongside the expansion of international amphibian trade [39].

The project has advanced understanding of wildlife disease ecology by providing empirical support for theoretical predictions that highly pathogenic pathogens can regulate host populations even at low prevalence [40,41] insights into host compensatory responses to pathogen pressure [42]; and evidence of highly localised transmission dynamics, demonstrating that observational scale shapes our ability to detect and interpret infection dynamics [40].

The project is founded on cross-sectoral collaboration, coordinated through the Binational Conservation Strategy for Darwin's Frogs [43], which has been led with long-term partners, including the Chilean NGO Ranita de Darwin (established as a direct outcome of this project), Universidad Andrés Bello, and Zoo Leipzig. This conservation strategy brings together governmental, non-profit, and private organisations to collaboratively implement priority actions for the conservation of Darwin's frogs and native forests in Chile and Argentina. Outcomes include Chile's formal implementation of amphibian disease reporting to the World Organisation for Animal Health, capacity-building in biosecurity, disease surveillance, and monitoring, and a rapid multi-partner response to Bd invasion in a key *R. darwinii* stronghold in Parque Tantauco [43].

In Parque Tantauco, we are implementing, together with the local partner Fundación Parque Tantauco, a field trial to evaluate an *in situ* Bd disease-mitigation intervention in *R. darwinii* (figure 2C). The response to Bd invasion in this private protected area also prompted emergency rescue actions to establish biosecure *ex situ* survival-assurance colonies in Chile and at ZSL, that will provide individuals for conservation reintroductions while also advancing understanding of chytridiomycosis and

supporting the development of mitigation strategies. Collectively, these activities form a multidisciplinary conservation response linking *in situ* and *ex situ* actions, exemplifying cross-institutional collaboration within ZSL and multi-partner collaboration beyond ZSL for wildlife health.

Bats and Bugs Project: interdisciplinary research in emerging pathogens in West Africa

Research into African fruit bats began in 2007, to identify and understand the ecology of bat-borne zoonotic viruses in Africa. Early studies detected antibodies to the rabies virus, Lagos bat virus (LBV), and to henipaviruses in multiple species, but particularly in the African straw-coloured fruit bat (*Eidolon helvum*; figure 2D) [44,45]. These results were significant because both types of virus can cause fatal human infections, this was the first evidence of henipavirus infection in the African continent, and rabies vaccine does not protect against LBV [44,45]. To further investigate these viruses, a long-term collaboration was established between ZSL, the University of Cambridge, the University of Ghana, and both the Veterinary Services Department and the Forestry Commission of the Government of Ghana [46].

The project employs a multidisciplinary approach, combining virology, genetics, and ecology to track pathogen circulation, and social sciences to understand human dimensions to zoonotic risk [47]. The previously underappreciated extent of the bat bushmeat trade in Ghana was uncovered [48], human behaviours that risk zoonotic transmission identified and public health mitigation strategies informed [49]. We showed *E. helvum* to be panmictic, thus the species exists as a single continent-wide epidemiological unit [46].

The establishment of a captive breeding colony of *E. helvum* in Accra in 2010 enabled longitudinal monitoring of a migratory bat species that is difficult to study in the wild [50]. Research using this colony demonstrated the long-term persistence of multiple paramyxoviruses [51], revealed ecological drivers of henipavirus seroprevalence [50], and enabled experimental Lagos bat virus infections in bats of known infection history, clarifying viral pathogenesis and shedding routes [52].

By identifying high-risk landscapes undergoing rapid land-use change and seasonal peaks in viral shedding [53], the project provides evidence-based strategies to support safe coexistence and mitigate pathogen spillover. This work promotes the conservation of African fruit bats, species which provide vital ecosystem services through pollination and seed dispersal, by demonstrating that human health and biodiversity protection are not mutually exclusive through the alignment of wildlife ecology with public health planning within a One Health framework.

Cetacean Strandings Investigation Programme: long-term national wildlife health surveillance

In 1988, a phocine distemper virus epidemic in European seals attracted widespread media and public attention, prompting the UK government to recognise the need for a systematic understanding of the causes of morbidity and mortality in marine mammals. This led to the creation of the ZSL-led Cetacean Strandings Investigation Programme (CSIP) in 1990, now funded by Defra and the Welsh Government to coordinate the investigation of Protected, Endangered and Threatened marine species found stranded along the coasts of England and Wales. Over the last 35 years it has recorded data on over 25,000 marine mammal strandings and conducted more than 3,500 post-mortem examinations (figure 2E), generating an internationally significant dataset and national tissue archive. Consistently gathered over decades, these tissues and data represent one of the world's most comprehensive wildlife health resources, providing a powerful baseline for detecting emerging threats.

Since its inception, the CSIP has described the first mass mortality event related to bycatch (accidental capture in fishing gear) in Europe [54], identified a condition in cetaceans analogous to decompression sickness [55], and provided some of the first evidence of fatal interactions between bottlenose dolphins and other cetaceans [56]. Long-term collaboration with the Centre for Environment, Fisheries and Aquaculture Science has produced the world's largest dataset on chemical pollutant exposure in marine mammals, underpinning globally significant research on toxicological impacts (e.g. [57]). The CSIP also led investigations into the phocine distemper virus

epidemic in 2002-2003 [58] and multiple mass stranding events, including some linked to anthropogenic noise [59]. CSIP data has highlighted how mobile marine species can act as sentinels of climate change [60] and the potential interaction between shifts in distribution and chemical pollution impacts [61].

Data collected by the CSIP directly support UK Marine Strategy reporting and contribute to international frameworks, including the International Whaling Commission and the Oslo–Paris Commission. Its evidence has driven key policy actions, such as the UK and EU bans on brominated flame retardants in the early 2000s and the 2004 ban on pair trawling in southwest waters. Sustained for over three decades, the CSIP exemplifies how long-term surveillance can deliver internationally relevant science while informing conservation.

Bovine tuberculosis management in the United Kingdom

The management of European badgers (*Meles meles*) to control bovine tuberculosis (bTB) in UK cattle has been controversial for half a century and, for almost as long, ZSL has been applying science to try to resolve the issue.

Although bTB is primarily a cattle disease, it is zoonotic, and the UK government is committed to eliminating it. Herds are tested regularly, test-positive animals are slaughtered, and affected farms are restricted from trading. Some herds stay under restriction for years, damaging farm businesses and impacting farmers' mental health.

By 1970, cattle-focused controls had eliminated bTB from most of Britain, but its persistence in a few areas suggested wildlife involvement. The first infected badger was found in 1971. Badger culling followed, and was immediately controversial [62]. In 1979 Lord Zuckerman, then ZSL President, led an independent review which identified badgers as a bTB reservoir, and recommended that culling should continue [63].

Over the next two decades badger culling failed to prevent the re-emergence of bTB across Britain. Eventually, the government launched the Randomised Badger Culling

Trial (RBCT), overseen by independent experts (including a ZSL researcher), to quantify the role of badger culling in controlling bTB in cattle [64].

The RBCT showed that, while large-scale badger culling could reduce cattle bTB, its impacts on badgers undermined its utility. Surviving badgers ranged more widely, mixing with one another and spreading infection [65–67]. Although badger numbers fell, the proportion with *M. bovis* rose, preventing the local elimination of bTB [66,68]. Major reductions in badger numbers could offset this rise in prevalence, and cattle bTB incidence fell inside large culling areas. But where fewer badgers were killed –in small-scale culls or around the margins of large culls– cattle bTB risks rose rather than falling [69,70]. Subsequent molecular analyses showed that most cattle were infected by other cattle, not badgers [71].

Despite warnings that badger culling could never eliminate bTB, farmers were licensed to cull on a massive scale. ZSL established the Cornwall Badger Project (figure 2F) to explore non-lethal alternatives, including farm husbandry [72,73], and badger vaccination. In a small pilot area, it showed that vaccination appeared not only technically effective (with 73% of badgers vaccinated and test-prevalence falling from 16% to 0% over four years), but also popular with local farmers [74]. These promising findings inspired an ongoing project, implemented jointly with the National Farmers Union, which seeks to replicate this initial success on a larger scale.

This work has fed into policy change. Badger culling has always been unpopular with the public, and the growing recognition that alternatives are available has helped Ministers to champion its end. In 2026, ZSL was part of the group which co-authored a new strategy for England, mapping out plans for bTB elimination focused primarily on controlling cattle-to-cattle spread, supplemented by targeted badger vaccination and without badger culling [75].

Code

A.V-S prepared all code for the analyses and wrote this Supplementary Material with assistance from ChatGPT 5.1. All interpretations, decisions, and final edits reflect the authors’ own judgement. Code and data to replicate our analysis are available at Zenodo here.

References

1. Pennington J, Socher R, Manning C. 2014 GloVe: Global Vectors for Word Representation. In *Proceedings of the 2014 Conference on Empirical Methods in Natural Language Processing (EMNLP)* (eds A Moschitti, B Pang, W Daelemans), pp. 1532–1543. Doha, Qatar: Association for Computational Linguistics. (doi:10.3115/v1/D14-1162)

2. Mikolov T, Chen K, Corrado G, Dean J. 2013 Efficient estimation of word representations in vector space. *arXiv* (doi:10.48550/arXiv.1301.3781)

3. Levy O, Goldberg Y. 2014 Neural word embedding as implicit matrix factorization. pp. 2177–2185.

4. Baroni M, Dinu G, Kruszewski G. 2014 Don't count, predict! A systematic comparison of context-counting vs. context-predicting semantic vectors. pp. 238–247. Baltimore, Maryland: Association for Computational Linguistics. (doi:10.3115/v1/P14-1023)

5. Jolliffe IT. 2002 *Principal Component Analysis*. New York: Springer. (doi:10.1007/b98835)

6. Manning CD, Raghavan P, Schütze H. 2008 *Introduction to Information Retrieval*. Cambridge: Cambridge University Press. (doi:10.1017/CBO9780511809071)

7. Spärck Jones K. 1972 A statistical interpretation of term specificity and its application in retrieval. *Journal of Documentation* **28**, 11–21. (doi:10.1108/eb026526)

8. Salton G, Buckley C. 1988 Term-weighting approaches in automatic text retrieval. *Information Processing & Management* **24**, 513–523. (doi:10.1016/0306-4573(88)90021-0)

9. Arora S, Liang Y, Ma T. 2017 A simple but tough-to-beat baseline for sentence embeddings. Toulon, France.

10. Clarivate. 2025 Web of Science Core Collection Search Fields. *Web of Science Support*. See https://webofscience.zendesk.com/hc/en-us/articles/26916258216209-Web-of-Science-Core-Collection-Search-Fields (accessed on 22 June 2026).

11. Clarivate. 2026 Web of Science Core Collection Advanced Search Field Tags. *Web of Science Support*. See https://webofscience.zendesk.com/hc/en-us/articles/26916347018257-Web-of-Science-Core-Collection-Advanced-Search-Field-Tags (accessed on 22 June 2026).

12. OpenAI. 2024 Vector embeddings. *OpenAI API documentation*. See https://developers.openai.com/api/docs/guides/embeddings (accessed on 22 June 2026).

13. Grootendorst M. 2022 BERTopic: Neural topic modeling with a class-based TF-IDF procedure. *arXiv* (doi:10.48550/arXiv.2203.05794)

14. McInnes L, Healy J, Saul N, Großberger L. 2018 UMAP: Uniform Manifold Approximation and Projection. *Journal of Open Source Software* **3**, 861. (doi:10.21105/joss.00861)

15. McInnes L, Healy J, Astels S. 2017 hdbscan: Hierarchical density based clustering. *Journal of Open Source Software* **2**, 205. (doi:10.21105/joss.00205)

16. Mu Y, Dong C, Bontcheva K, Song X. 2024 Large Language Models Offer an Alternative to the Traditional Approach of Topic Modelling. pp. 10160–10171. Torino, Italia: ELRA and ICCL. (doi:10.18653/v1/2024.lrec-main.887)

17. Murray D, Ni C, Hubbard T. 2026 Using language models to label clusters of scientific documents. *Scientometrics* **131**, 2303–2332. (doi:10.1007/s11192-025-05445-5)

18. Cunningham AA. 1996 Disease risks of wildlife translocations. *Conserv. Biol.* **10**, 349–353. (doi:10.1046/j.1523-1739.1996.10020349.x)

19. Lawson B, Howard T, Kirkwood JK, Macgregor SK, Perkins M, Robinson RA, Ward LR, Cunningham AA. 2010 Epidemiology of salmonellosis in garden birds in England and Wales, 1993 to 2003. *EcoHealth* **7**, 294–306. (doi:10.1007/s10393-010-0349-3)

20. Hanmer HJ, Cunningham AA, John SK, Magregor SK, Robinson RA, Seilern-Moy K, Siriwardena GM, Lawson B. 2022 Habitat-use influences severe disease-mediated population declines in two of the most common garden bird species in Great Britain. *Sci Rep* **12**, 15055. (doi:10.1038/s41598-022-18880-8)

21. Cunningham AA, Smith F, McKinley TJ, Perkins MW, Fitzpatrick LD, Wright ON, Lawson B. 2019 Apparent absence of *Batrachochytrium salamandrivorans* in wild urodeles in the United Kingdom. *Sci. Rep.* **9**, 2831. (doi:10.1038/s41598-019-39338-4)

22. Franklinos LHV *et al.* 2017 Emerging fungal pathogen *Ophidiomyces ophiodiicola* in wild European snakes. *Sci. Rep.* **7**, 3844. (doi:10.1038/s41598-017-03352-1)

23. Folly AJ *et al.* 2020 Detection of Usutu virus infection in wild birds in the United Kingdom, 2020. *Euro Surveill* **25**, 2001732. (doi:10.2807/1560-7917.ES.2020.25.41.2001732)

24. Seilern-Macpherson K, Lawson B, Charman S, Jones S, Rainford J, Macarthur R, Kent AJ, Cunningham AA, Barnett L. 2026 Exposure to second-generation anticoagulant rodenticides is widespread in the non-target British wild mammals

Erinaceus europaeus and Vulpes vulpes. *Science of The Total Environment* **1043**, 181927. (doi:10.1016/j.scitotenv.2026.181927)

25. Lawson B *et al.* 2018 Salmonella Enteritidis ST183: emerging and endemic biotypes affecting western European hedgehogs (*Erinaceus europaeus*) and people in Great Britain. *Sci. Rep.* **8**, 2449. (doi:10.1038/s41598-017-18667-2)

26. Bengtsson RJ *et al.* 2023 The genomic epidemiology of *Escherichia albertii* infecting humans and birds in Great Britain. *Nat Commun* **14**, 1707. (doi:10.1038/s41467-023-37312-3)

27. Department for Environment, Food & Rural Affairs. 2025 Wild bird populations in the UK and England, 1970 to 2024.

28. Joyce L, Spiro S, Diener E, Stadler S, Guthrie A. 2025 Retrospective mortality review of Socorro doves (*Zenaida graysoni*) in zoological institutions worldwide: 1993–may 2024. *J. Zoo Wildl. Med.* **56**, 725–734. (doi:10.1638/2024-0135)

29. Sando A, Stidworthy MF, Rich AF, Wrigglesworth E, Blake DP, Strike T, Spiro S. 2025 Angiostrongylus vasorum in captive meerkats (*Suricata suricatta*) in the United Kingdom: significance, histopathology, and association with pulmonary arterial atherosclerosis. *J. Zoo Wildl. Med.* **56**, 756–767. (doi:10.1638/2024-0102)

30. Spiro S, Griffiths A, Arnaout A, Wrigglesworth E, Macgregor SK, John SK, Tahas SA, Nye E, Morrell AP. 2025 Hemosiderosis is associated with increased susceptibility to *Yersinia pseudotuberculosis* infection in Seba's short-tailed bats (*Carollia perspicillata*). *Vet Pathol* **62**, 1024–1036. (doi:10.1177/03009858251343017)

31. Boch M *et al.* 2026 Between-species variation in neocortical sulcal anatomy of the carnivoran brain. *eLife* **13**, RP100851. (doi:10.7554/eLife.100851)

32. Meredith A *et al.* 2022 Capacity building for wildlife health professionals: the Wildlife Health Bridge. *OHIR* , 68–78. (doi:10.20517/ohir.2022.03)

33. Tapley B, Rendle M, Baines FM, Goetz M, Bradfield KS, Rood D, Lopez J, Garcia G, Routh A. 2015 Meeting ultraviolet B radiation requirements of amphibians in captivity: A case study with mountain chicken frogs (*Leptodactylus fallax*) and general recommendations for pre-release health screening. *Zoo Biology* **34**, 46–52. (doi:10.1002/zoo.21170)

34. Flach EJ *et al.* 2024 Five years of Partula snail pre-export health screening. *J. Zoo Wildl. Med.* **55**, 31–41. (doi:10.1638/2023-0077)

35. Gurnell J, Reeve N, Cross B. 2026 On the Brink, a population of hedgehogs in Central London. *Ecol. Evol.* **16**, e72867. (doi:10.1002/ece3.72867)

36. Soto-Azat C, Valenzuela-Sánchez A, Collen B, Rowcliffe JM, Veloso A, Cunningham AA. 2013 The population decline and extinction of Darwin's frogs. *PLOS ONE* **8**, e66957. (doi:10.1371/journal.pone.0066957)

37. Soto-Azat C, Valenzuela-Sánchez A, Clarke BT, Busse K, Ortiz JC, Barrientos C, Cunningham AA. 2013 Is Chytridiomycosis driving Darwin's frogs to extinction? *PLOS ONE* **8**, e79862. (doi:10.1371/journal.pone.0079862)

38. Valenzuela-Sánchez A, O'Hanlon SJ, Alvarado-Rybak M, Uribe-Rivera DE, Cunningham AA, Fisher MC, Soto-Azat C. 2018 Genomic epidemiology of the emerging pathogen *Batrachochytrium dendrobatidis* from native and invasive amphibian species in Chile. *Transbound. Emerg. Dis.* **65**, 309–314. (doi:10.1111/tbed.12775)

39. O'Hanlon SJ *et al.* 2018 Recent Asian origin of chytrid fungi causing global amphibian declines. *Science* **360**, 621–627. (doi:10.1126/science.aar1965)

40. Valenzuela-Sánchez A *et al.* 2026 Localized transmission of an aquatic pathogen drives hidden epidemics and population collapse in a terrestrial host. *Nat. Ecol. Evol.* **10**, 308–317. (doi:10.1038/s41559-025-02930-1)

41. Valenzuela-Sánchez A, Schmidt BR, Uribe-Rivera DE, Costas F, Cunningham AA, Soto-Azat C. 2017 Cryptic disease-induced mortality may cause host extinction in an apparently stable host–parasite system. *Proc. Biol. Sci.* **284**, 20171176. (doi:10.1098/rspb.2017.1176)

42. Valenzuela-Sánchez A *et al.* 2022 Interpopulation differences in male reproductive effort drive the population dynamics of a host exposed to an emerging fungal pathogen. *J Anim Ecol* **91**, 308–319. (doi:10.1111/1365-2656.13603)

43. Valenzuela-Sánchez A *et al.* 2026 Estrategia Binacional de Conservación de las Ranitas de Darwin (2a ed).

44. Hayman DTS, Suu-Ire R, Breed AC, McEachern JA, Wang L, Wood JLN, Cunningham AA. 2008 Evidence of Henipavirus infection in West African fruit bats. *PLOS ONE* **3**, e2739. (doi:10.1371/journal.pone.0002739)

45. Hayman DTS, Fooks AR, Horton D, Suu-Ire R, Breed AC, Cunningham AA, Wood JLN. 2008 Antibodies against lagos bat virus in Megachiroptera from West Africa. *Emerg. Infect. Dis.* **14**, 926–928. (doi:10.3201/eid1406.071421)

46. Peel AJ *et al.* 2013 Continent-wide panmixia of an African fruit bat facilitates transmission of potentially zoonotic viruses. *Nat. Commun.* **4**, 2770. (doi:10.1038/ncomms3770)

47. Wood JLN *et al.* 2012 A framework for the study of zoonotic disease emergence and its drivers: spillover of bat pathogens as a case study. *Philos. Trans. R. Soc. Lond. B Biol. Sci.* **367**, 2881–2892. (doi:10.1098/rstb.2012.0228)

48. Kamins AO, Restif O, Ntiamoa-Baidu Y, Suu-Ire R, Hayman DTS, Cunningham AA, Wood JLN, Rowcliffe JM. 2011 Uncovering the fruit bat bushmeat commodity chain and the true extent of fruit bat hunting in Ghana, West Africa. *Biol. Conserv.* **144**, 3000–3008. (doi:10.1016/j.biocon.2011.09.003)

49. Kamins AO, Rowcliffe JM, Ntiamoa-Baidu Y, Cunningham AA, Wood JLN, Restif O. 2015 Characteristics and risk perceptions of ghanaians potentially exposed to bat-borne zoonoses through bushmeat. *EcoHealth* **12**, 104–120. (doi:10.1007/s10393-014-0977-0)

50. Baker KS *et al.* 2014 Viral antibody dynamics in a chiropteran host. *J Anim Ecol* **83**, 415–428. (doi:10.1111/1365-2656.12153)

51. Gibson L, Ribas MP, Kemp J, Restif O, Suu-Ire RD, Wood JLN, Cunningham AA. 2021 Persistence of Multiple Paramyxoviruses in a Closed Captive Colony of Fruit Bats (*Eidolon helvu*m). *Viruses* **13**. (doi:10.3390/v13081659)

52. Begeman L *et al.* 2020 Experimental Lagos bat virus infection in straw-colored fruit bats: A suitable model for bat rabies in a natural reservoir species. *PLOS Negl. Trop. Dis.* **14**, e0008898. (doi:10.1371/journal.pntd.0008898)

53. Jolma ER, Gibson L, Suu-Ire RD, Fleischer G, Asumah S, Languon S, Restif O, Wood JLN, Cunningham AA. 2021 Longitudinal secretion of Paramyxovirus RNA in the urine of straw-coloured fruit bats (*Eidolon helvum*). *Viruses* **13**. (doi:10.3390/v13081654)

54. Kuiken T *et al.* 1994 Mass mortality of common dolphins (*Delphinus delphis*) in South West England due to incidental capture in fishing gear. *Vet Rec* **134**, 81–89. (doi:10.1136/vr.134.4.81)

55. Jepson PD *et al.* 2003 Gas-bubble lesions in stranded cetaceans. *Nature* **425**, 575–576. (doi:10.1038/425575a)

56. Barnett J, Davison N, Deaville R, Monies R, Loveridge J, Tregenza N, Jepson PD. 2009 Postmortem evidence of interactions of bottlenose dolphins (Tursiops truncatus) with other dolphin species in south-west England. *Vet Rec* **165**, 441–444. (doi:10.1136/vr.165.15.441)

57. Williams RS *et al.* 2023 Spatiotemporal Trends Spanning Three Decades Show Toxic Levels of Chemical Contaminants in Marine Mammals. *Environ. Sci. Technol.* **57**, 20736–20749. (doi:10.1021/acs.est.3c01881)

58. Lawson B, Jepson PD. 2003 Investigation of a PDV Epizootic in the UK during 2002/03.

59. Jepson PD *et al.* 2013 What caused the UK's largest common dolphin (Delphinus delphis) mass stranding event? *PLOS ONE* **8**, e60953. (doi:10.1371/journal.pone.0060953)

60. Williamson MJ, ten Doeschate MTI, Deaville R, Brownlow AC, Taylor NL. 2021 Cetaceans as sentinels for informing climate change policy in UK waters. *Mar. Policy.* **131**, 104634. (doi:10.1016/j.marpol.2021.104634)

61. Williams RS *et al.* 2025 Sea temperature and pollution are associated with infectious disease mortality in short-beaked common dolphins. *Commun Biol* **8**, 557. (doi:10.1038/s42003-025-07858-7)

62. Cassidy A. 2019 *Vermin, Victims and Disease: British Debates over Bovine Tuberculosis and Badgers*. Cham, Switzerland: Springer International Publishing. (doi:10.1007/978-3-030-19186-3)

63. Zuckerman O. 1980 Badgers, cattle and tuberculosis. , 107.

64. Krebs JR, Anderson RM, Clutton-Brock T, Donnelly CA, Frost S, Morrison WI, Woodroffe R, Young D. 1998 Badgers and bovine TB: conflicts between conservation and health. *Science* **279**, 817–818. (doi:10.1126/science.279.5352.817)

65. Woodroffe R, Donnelly CA, Cox DR, Bourne FJ, Cheeseman CL, Delahay RJ, Gettinby G, Mcinerney JP, Morrison WI. 2006 Effects of culling on badger (*Meles meles*) spatial organization: implications for the control of bovine tuberculosis. *J. Appl. Ecol.* **43**, 1–10. (doi:10.1111/j.1365-2664.2005.01144.x)

66. Woodroffe R *et al.* 2006 Culling and cattle controls influence tuberculosis risk for badgers. *PNAS* **103**, 14713–14717. (doi:10.1073/pnas.0606251103)

67. Ham C, Donnelly CA, Astley KL, Jackson SYB, Woodroffe R. 2019 Effect of culling on individual badger (*Meles meles*) behaviour: Potential implications for bovine tuberculosis transmission. *J. Appl. Ecol.* **56**, 2390–2399. (doi:10.1111/1365-2664.13512)

68. Woodroffe R *et al.* 2009 Bovine tuberculosis in cattle and badgers in localized culling areas. *J. Wildl. Dis.* **45**, 128–143. (doi:10.7589/0090-3558-45.1.128)

69. Donnelly CA, Woodroffe R, Cox DR, Bourne J, Gettinby G, Le Fevre AM, McInerney JP, Morrison WI. 2003 Impact of localized badger culling on tuberculosis incidence in British cattle. *Nature* **426**, 834–837. (doi:10.1038/nature02192)

70. Donnelly CA *et al.* 2006 Positive and negative effects of widespread badger culling on tuberculosis in cattle. *Nature* **439**, 843–846. (doi:10.1038/nature04454)

71. van Tonder AJ *et al.* 2021 Inferring Mycobacterium bovis transmission between cattle and badgers using isolates from the randomised badger culling trial. *PLOS Pathogens* **17**, e1010075. (doi:10.1371/journal.ppat.1010075)

72. Woodroffe R, Donnelly CA, Ham C, Jackson SYB, Moyes K, Chapman K, Stratton NG, Cartwright SJ. 2016 Badgers prefer cattle pasture but avoid cattle: implications for bovine tuberculosis control. *Ecol. Lett.* **19**, 1201–1208. (doi:10.1111/ele.12654)

73. Woodroffe R, Donnelly CA, Ham C, Jackson SYB, Moyes K, Chapman K, Stratton NG, Cartwright SJ. 2016 Use of farm buildings by wild badgers: implications for the transmission of bovine tuberculosis. *Eur. J. Wildl. Res.* **63**, 6. (doi:10.1007/s10344-016-1065-2)

74. Woodroffe R *et al.* 2024 Farmer-led badger vaccination in Cornwall: Epidemiological patterns and social perspectives. *People Nat.* **6**, 1960–1973. (doi:10.1002/pan3.10691)

75. Cross J *et al.* 2026 Bovine TB control strategy for England: As recommended by the Steering Group of the Bovine TB Partnership for England.

Tables

Table S1. Included and excluded seed terms used to characterise the health concept. Terms were retained only if they were available in the embedding models and met minimum frequency thresholds (≥10 occurrences and ≥0.1% of documents).

Status	Seeds
Included	wildlife, health, zoonotic, spillover, zoonosis, zoonoses, pathogen, biodiversity, conservation, surveillance, veterinary, fitness, adaptation, epidemic, pandemic, epizootic, disease, physical, behavioural, behavioral, social, resilience, change, ecosystem, population, individual, biological, environmental, determinants, stability, anthropogenic, goals, capacity, state, reproduction, welfare, wellbeing, persist, persistence
Excluded	illness, panzootic, biologic, renewal, selfrenewal, cope, expectations, construct, mental, infirmity, psychological, productivity, recover, ethics

Table S2. Conceptual domains identified from the wildlife–health corpus, with their ten most representative terms from the GloVe and word2vec embedding models, the number of terms assigned to each domain in both models, and the mean semantic share (GloVe) across all documents.

Domain	Top 10 GloVe Terms	Top 10 word2vec Terms	Domain Size (GloVe / w2v)	Mean Semantic Share (GloVe)
Infection and disease ecology	wildlife, disease, health, diseases, human, conservation, impact, zoonotic, risk, environmental	fever, disease, swine, francisella, acari, zoonotic, ehrlichia, diseases, hemorrhagic, infectious	63 / 126	0.498
Monitoring, management, and policy	ecosystem, monitoring, national, center, veterinary, research, capacity, integrated, ecological, medicine	governance, interdisciplinary, health, sciences, wildlife, curriculum, plans, implement, initiatives, management	113 / 143	0.231
Ecological responses	salmonella, goals, distance, iron, persistence, costs, biotic, young, shot, allows	stressors, physiological, immune, traits, fitness, reproductive, physiology, disturbances, behavioural, urbanization	256 / 177	0.271

Table S3. Summary of semantic topic clusters identified from ZSL publications. Keywords are short terms chosen by a GPT-4–class model to summarise the dominant themes, taxa, processes, or methods represented in that cluster, based on a sample of titles and abstracts. Topics in the Wildlife health category are shown in bold letters, and a description of why each topic was manually classified as belonging to this category is provided in the Description column.

Topic label	Description	Keywords	N papers
Marine Biodiversity and Conservation Ecology	This cluster focuses on the study of marine ecosystems, biodiversity patterns, and conservation strategies across various taxa including fishes, sharks, seahorses, and benthic invertebrates. Research addresses ecological dynamics, habitat use, human impacts, and governance of marine protected areas using field observations, tracking technologies, and ecological assessments in diverse oceanic regions. The work aims to inform effective management and conservation of vulnerable marine species and habitats under environmental and anthropogenic pressures.	marine biodiversity; conservation biology; reef ecology; marine protected areas; species monitoring	206
**Marine Mammal Health and Conservation**	This cluster focuses on the health, disease epidemiology, population dynamics, and conservation challenges of marine mammals, particularly cetaceans and pinnipeds. Studies investigate impacts of infectious diseases, pollutants like PCBs, climate change effects on distribution, and population declines using necropsies, genetic analyses, epidemiological surveys, and long-term monitoring. *Why wildlife health?:* Addresses disease epidemiology, pollutant impacts, nutritional status, genetic population structure, and conservation challenges affecting marine mammals, which are critical for understanding and managing population health and declines.	marine mammals; disease epidemiology; pollution impact; population decline; climate change	184
**Amphibian Infectious Diseases and Ecology**	This cluster focuses on the study of emerging infectious diseases affecting amphibian populations worldwide, particularly fungal, viral, and parasitic pathogens such as Batrachochytrium dendrobatidis and ranaviruses. Research addresses disease dynamics, host-pathogen interactions, environmental influences, and diagnostic methods to understand impacts on amphibian biodiversity and inform conservation strategies. *Why wildlife health?:* Addresses the emergence, transmission, host–	amphibians; infectious disease; Batrachochytrium dendrobatidis; ranavirus; wildlife epidemiology	177

Topic label	Description	Keywords	N papers
	pathogen interactions, environmental influences, and impacts of fungal and viral infectious diseases in amphibian populations, which are central to understanding and mitigating global biodiversity loss.		
**Semen Cryopreservation and Sperm Biology**	This cluster focuses on the cryopreservation, physiology, and functional biology of spermatozoa across various taxa including mammals and fish. Research addresses mechanisms of sperm motility, DNA integrity, fertilization processes, and the development of improved semen preservation techniques using molecular, cellular, and cryobiological methods. *Why wildlife health?:* Focuses on sperm cryopreservation and reproductive biology relevant to genetic conservation, fertility management, and the maintenance of adaptive potential across wildlife species.	sperm cryopreservation; semen technology; sperm motility; DNA fragmentation; reproductive biology	174
Global Vertebrate Conservation and Extinction	This cluster focuses on assessing the conservation status, extinction risks, and evolutionary significance of vertebrate species worldwide. It integrates phylogenetic metrics, historical data, and IUCN Red List assessments to prioritise conservation efforts and understand patterns of population decline and resilience. Taxa covered include mammals, birds, amphibians, reptiles, fishes, and deep-sea molluscs, with methods spanning ecological modelling, phylogenetics, and data synthesis to inform global biodiversity conservation strategies.	conservation status; extinction risk; phylogenetic diversity; IUCN Red List; vertebrates	138
Primate Social Behavior and Reproduction	This cluster investigates the social dynamics, mating strategies, and cognitive behaviors of wild and captive primates. Studies employ behavioral observations, experimental tasks, and genetic analyses to explore sexual coercion, mate choice, social network structure, and reproductive outcomes. The research aims to understand evolutionary and ecological drivers of social and reproductive behaviors in primates.	primate behavior; sexual selection; social networks; mate choice; reproductive strategies	134
**Reproductive Endocrinology in Mammals**	This cluster focuses on the hormonal regulation of reproduction across various mammalian species. Studies investigate steroid hormone patterns, ovulation induction, superovulation, follicular development, and endocrine control mechanisms using radioimmunoassay, in vitro culture,	reproductive endocrinology; ovulation; steroid hormones; assisted reproduction; mammals	126

Topic label	Description	Keywords	N papers
	hormone treatments, and embryo transfer. *Why wildlife health?:* Investigates hormonal regulation, reproductive physiology, and assisted reproduction techniques essential for managing fertility, breeding programmes, and the conservation of mammalian wildlife.		
Amphibian and Reptile Taxonomy and Conservation	This cluster focuses on the taxonomy, systematics, and conservation biology of amphibians and reptiles from diverse geographic regions. Studies employ morphological, molecular, and ecological methods to describe new species, clarify taxonomic statuses, and investigate conservation challenges such as habitat degradation and captive breeding. The research aims to enhance understanding of species diversity and inform conservation strategies for endangered and microendemic taxa.	taxonomy; amphibians; reptiles; conservation; molecular phylogenetics	106
**Avian Infectious Diseases and Pathology**	This cluster focuses on the diagnosis, epidemiology, and pathological impacts of infectious diseases and toxicological conditions affecting wild and captive bird species. Research includes bacterial, viral, and parasitic infections, and heavy metal toxicosis, employing molecular diagnostics, histopathology, and epidemiological surveillance. *Why wildlife health?:* Examines infectious diseases, toxicological conditions, and pathological abnormalities affecting wild and captive birds in Great Britain, using diagnostic, molecular, and epidemiological approaches to assess disease prevalence, transmission, and conservation impacts.	avian diseases; wild birds; pathology; epidemiology; molecular diagnostics	106
Invasive Alien Species Impacts and Management	This cluster focuses on ecological impacts, spread dynamics, and risk assessment of invasive alien species. It explores species distribution models, expert elicitation (EICAT), and horizon scanning to predict invasions and inform conservation translocations and biosecurity.	invasive alien species; species distribution models; EICAT; biosecurity; conservation translocation	105
Genetic Analysis in Wildlife Populations	This cluster focuses on genetic methods and analyses used to study population structure, relatedness, and genetic diversity in wild animal populations. It addresses challenges such as genotyping errors and sample misidentification, employing molecular markers and computational tools to infer sibship, admixture, inbreeding, and migration. The research aims to improve conservation strategies by monitoring	genetic markers; population structure; sibship reconstruction; genotyping errors; conservation genetics	104

Topic label	Description	Keywords	N papers
	genetic erosion and understanding evolutionary and ecological dynamics in species like bats, sea lions, and other wildlife.		
Conservation and Ecology of African Carnivores	This cluster focuses on the ecology, population dynamics, conservation challenges, and human-wildlife interactions of African large carnivores, particularly cheetahs and leopards. Studies employ methods such as camera trapping, occupancy modelling, demographic analysis, and trade route mapping to understand species abundance, habitat use, reproductive success, and threats including illegal trade and human conflict across various African landscapes. The research aims to inform conservation strategies for vulnerable carnivore populations in human-disturbed and protected areas.	cheetah ecology; large carnivore conservation; population dynamics; habitat use; illegal wildlife trade	90
**Wildlife Disease Ecology and Management**	This cluster focuses on understanding the dynamics, risks, and management of infectious diseases in wildlife and their implications for conservation, public health, and ecosystem stability. It covers zoonotic spillover, disease surveillance, vector ecology, and biosecurity in species translocations, using integrative modelling, epidemiological frameworks, and multidisciplinary approaches. *Why wildlife health?:* Addresses the dynamics, risks, surveillance, and management of infectious diseases in wildlife populations, including zoonotic spillover and implications for conservation, public health, and ecosystem stability.	zoonoses; wildlife disease surveillance; disease ecology; biosecurity; One Health	87
Essential Fatty Acids in Mammalian Biology	This cluster investigates the metabolism, composition, and developmental roles of essential fatty acids, particularly in mammals including humans, cats, dolphins, and rodents. Research focuses on dietary intake, lipid incorporation in brain and liver tissues, and comparative analyses across species to understand evolutionary and physiological implications. Methods include biochemical assays, dietary surveys, and comparative lipid profiling in different ecological and socioeconomic contexts.	essential fatty acids; lipid metabolism; mammalian nutrition; brain development; comparative biochemistry	83
Pangolin Ecology and Conservation Biology	This cluster focuses on the ecology, genetics, monitoring, and conservation of pangolin species across Asia and Africa. Research addresses dietary habits, genetic diversity, tracking methodologies, reproductive biology,	Pangolin ecology; Conservation genetics; Wildlife monitoring;	81

Topic label	Description	Keywords	N papers
	and the impact of illegal trade, employing field studies, genetic analyses, and ecological monitoring techniques. The work aims to inform conservation strategies and improve species management under threats from poaching and habitat loss.	Illegal wildlife trade; Captive breeding	
Serological Diagnostics of Tropical Parasites	This cluster focuses on the development and evaluation of serological assays, particularly enzyme-linked immunosorbent assays (ELISA) and related immunoassays, for detecting antibodies and antigens of tropical parasitic diseases. The research covers a range of pathogens including Trypanosoma species, Plasmodium (malaria), Entamoeba histolytica, and Leishmania, using samples from humans and animal models. The studies aim to improve diagnostic specificity and sensitivity to aid disease management and epidemiological surveillance in tropical regions.	ELISA; tropical parasitic diseases; serodiagnosis; Trypanosoma; malaria	77
Social Evolution and Conservation of Insects	This cluster explores the social structure, reproductive strategies, and ecological roles of eusocial insects such as ants, bees, and wasps. Studies investigate kin selection, reproductive skew, division of labour, phenotypic plasticity, and foraging behavior using field observations, molecular analyses, and theoretical models. Conservation genetics and habitat impacts on pollinator species are also addressed to inform biodiversity preservation.	eusocial insects; reproductive skew; kin selection; phenotypic plasticity; conservation genetics	67
**Canid Disease Ecology and Conservation**	This cluster explores infectious disease dynamics, immune genetics, and conservation challenges in wild and domestic canids, including African wild dogs, Ethiopian wolves, and rural domestic dogs. Studies employ serosurveys, molecular diagnostics, population genetics, and vaccination trials to understand pathogen transmission, immune variation, and impacts on endangered canid populations in diverse geographic contexts. *Why wildlife health?:* Focuses on infectious diseases affecting wild and domestic canids, including transmission dynamics, host interactions, and conservation consequences for endangered species such as African wild dogs and Ethiopian wolves.	canids; infectious disease; rabies; canine distemper virus; conservation biology	64
Global Biodiversity Conservation Strategies	This cluster focuses on the challenges and strategies for conserving biodiversity worldwide, addressing	biodiversity conservation; ecological data;	61

Topic label	Description	Keywords	N papers
	issues such as biodiversity loss, conservation policy, and the impact of human activities. It emphasizes interdisciplinary collaboration, communication between scientists and practitioners, and the use of ecological data to inform conservation efforts across multiple taxa. The research also explores international frameworks and targets for biodiversity protection and recovery.	conservation policy; wildlife crime; international frameworks	
Satellite Remote Sensing in Biodiversity	This cluster explores the application of satellite remote sensing technologies to monitor and manage biodiversity, ecosystems, and natural resources. It addresses challenges in assessing species diversity, ecosystem extent, and environmental pressures, emphasizing the integration of remote sensing data in conservation policy and ecosystem risk assessments.	satellite remote sensing; biodiversity monitoring; ecosystem assessment; natural resource management; conservation policy	60
Global Biodiversity Conservation and Monitoring	This cluster focuses on assessing global biodiversity trends, conservation targets, and policy frameworks. It addresses methods for monitoring species populations, evaluating conservation effectiveness, and integrating biodiversity indicators to guide international conservation efforts, spanning multiple taxa and emphasising vertebrates and megafauna.	biodiversity monitoring; conservation policy; global biodiversity targets; species population trends; biodiversity indicators	60
**Bat-Borne Viral Zoonoses Ecology**	This cluster investigates the ecology, transmission, and host dynamics of bat-borne viruses with zoonotic potential. Studies encompass viral detection, antibody prevalence, tissue receptor distribution, and genetic population structure, often employing serological assays, molecular diagnostics, and population genetics. *Why wildlife health?:* Examines the ecology, transmission, and zoonotic potential of bat-borne viruses, including lyssaviruses, henipaviruses, and filoviruses, with implications for bat population health and spillover risk to humans and domestic animals.	bat viruses; zoonoses; paramyxoviruses; filoviruses; lyssaviruses	59
Mangrove Conservation and Plastic Pollution	This cluster focuses on the conservation, management, and ecological importance of mangrove ecosystems, including stakeholder perceptions, restoration strategies, and impacts of anthropogenic threats such as microplastic pollution. Methods involve remote sensing, social network analysis, bibliometric reviews, and	mangroves; conservation; microplastics; stakeholder perceptions; remote sensing	57

Topic label	Description	Keywords	N papers
	pollution characterisation across diverse geographic contexts.		
Wildlife Reintroduction and Monitoring	This cluster focuses on the ecological and conservation challenges associated with reintroducing endangered species. It explores factors influencing reintroduction success such as release strategies, habitat selection, dispersal behavior, and invasive species management, using field experiments, population modelling, and long-term monitoring.	reintroduction; population monitoring; conservation biology; habitat selection; invasive species management	57
**Wildlife Veterinary Pathology and Diseases**	This cluster focuses on the pathological conditions, diseases, and anatomical studies of wild and captive mammals. Research includes diagnostic investigations of tumours, infections, deformities, and physiological adaptations using histology, immunohistochemistry, radiography, and clinical observations. *Why wildlife health?:* Addresses the diagnosis, pathology, treatment, and management of diseases and anatomical abnormalities in captive and wild mammals in zoological contexts, with direct implications for animal health and welfare.	wildlife pathology; veterinary medicine; mammalian diseases; diagnostic histology; zoological health	55
Gazelle Ecology and Conservation in Arabia	This cluster focuses on the ecology, population dynamics, and conservation of gazelle species in arid regions. Studies employ behavioural observations, population surveys, phylogenetic analyses, and habitat use assessments to understand feeding habits, social organisation, genetic diversity, and human impacts.	Gazella arabica; population dynamics; feeding ecology; conservation genetics; arid habitat	53
**Avian Reproduction and Conservation Biology**	This cluster focuses on reproductive biology, life-history traits, and conservation challenges in birds. Studies investigate reproductive success and genetic diversity using field data and experimental manipulations to inform conservation strategies. *Why wildlife health?:* Investigates avian reproductive biology, including factors influencing fertility, hatching failure, genetic diversity, and conservation strategies relevant to the persistence of threatened bird populations.	avian reproduction; hatching failure; sexual selection; genetic diversity; conservation biology	51
Body Size, Ecology, and Metabolic Scaling	This cluster investigates how body size influences ecological traits, metabolic rates, and life-history strategies across diverse vertebrates using comparative analyses, modelling, and large datasets.	body size; metabolic scaling; ecology; life history; predator-prey dynamics	50

Topic label	Description	Keywords	N papers
Bushmeat Hunting and Consumption Dynamics	This cluster investigates ecological, sociocultural, and economic aspects of bushmeat hunting and consumption. It explores impacts on wildlife populations, drivers behind consumer behaviour, and the role of bushmeat in livelihoods using surveys, interviews, and systematic reviews.	bushmeat hunting; wildlife conservation; consumer behavior; rural livelihoods; sustainable resource use	49
Camera Trapping for Wildlife Monitoring	This cluster focuses on camera traps and related technologies to monitor wildlife populations, estimate densities, and assess biodiversity. Research addresses methodological advancements, data management, and applications for elusive species.	camera traps; animal density estimation; wildlife monitoring; remote sensing; conservation biology	46
**Zoo Animal Management and Conservation**	This cluster focuses on management, breeding, and conservation of animals within zoological institutions, including husbandry and veterinary care, and collaborative efforts to conserve threatened species. *Why wildlife health?:* Addresses veterinary care, artificial breeding, and management strategies essential for the health, welfare, and conservation of zoo animals and threatened wildlife species.	zoo management; artificial breeding; wildlife conservation; veterinary care; species preservation	43
Animal Movement and Conservation Challenges	This cluster explores animal movement patterns and their implications for conservation planning, integrating statistical and technological approaches to understand responses to environmental change.	animal movement; conservation planning; migration; Asian elephants; behavioral ecology	41
Seabird Foraging and Migration Ecology	This cluster focuses on foraging behaviour, migration strategies, and spatial ecology of seabirds using tracking and biologging. Research addresses how environmental factors and individual traits influence movement and breeding performance.	seabird migration; foraging behavior; biologging; marine spatial ecology; breeding performance	41
**Botulism and Fusobacterium in Wildlife**	This cluster investigates the ecology, pathogenicity, and diagnosis of Clostridium botulinum and Fusobacterium necrophorum infections in wildlife, including environmental reservoirs and factors influencing toxicity. *Why wildlife health?:* Examines the ecology, pathogenicity, and epidemiology of Clostridium botulinum and Fusobacterium necrophorum, the causative agents of botulism and	Clostridium botulinum; Fusobacterium necrophorum; botulism; necrobacillosis; wildlife disease ecology	40

Topic label	Description	Keywords	N papers
	necrobacillosis, which affect waterfowl and other wildlife.		
Tropical Land Use and Biodiversity	This cluster investigates impacts of land use changes on tropical ecosystems and biodiversity, including agriculture, deforestation, and climate change interactions, using empirical and interdisciplinary approaches.	tropical deforestation; biodiversity; land use change; climate resilience; agroforestry	40
Vertebrate Blood Biochemistry and Physiology	This cluster focuses on biochemical and physiological properties of blood across vertebrates, addressing reference limits, plasma proteins, and pathological responses using biochemical measurement and comparative analyses.	blood biochemistry; vertebrates; hemoglobin; plasma proteins; vascular physiology	34
Conservation and Ecology of Anguillid Eels	This cluster focuses on ecology, behaviour, and conservation management of anguillid eels, including migration and interventions such as fish passage improvement and trap-and-transport.	European eel; Anguillid eels; conservation management; migration ecology; habitat connectivity	33
Conservation and Ecology of Gibbons	This cluster focuses on conservation biology, ecology, and monitoring of gibbons, including the Hainan gibbon, using acoustic analysis, drones, and niche modelling, and integrating traditional ecological knowledge.	Hainan gibbon; primate conservation; population monitoring; ecological niche modeling; traditional ecological knowledge	33
Marine Invertebrate Molecular Ecology	This cluster focuses on molecular phylogenetics, population genetics, and biodiversity assessment of marine invertebrates using sequencing, mitochondrial genome analyses, DNA barcoding, and eDNA approaches.	mitochondrial genomes; DNA barcoding; Pennatulacea; marine nematodes; environmental DNA	31
Biodiversity Monitoring and Spatial Ecology	This cluster focuses on methods and challenges in monitoring biodiversity using citizen science and spatial modelling, covering taxa such as bats, pollinators, butterflies, and ladybirds, and addressing data quality and environmental change.	citizen science; species distribution modeling; population monitoring; habitat diversity; pollinator biodiversity	29
**Zoo Animal Behavior and Welfare**	This cluster examines behaviour, welfare, and enrichment of zoo-housed animals, including visitor effects, personality, and technological	zoo animal behavior; animal welfare; enrichment;	29

Topic label	Description	Keywords	N papers
	monitoring methods to improve captive welfare. *Why wildlife health?:* Focuses on assessing and improving welfare, behaviour, and enrichment of zoo-housed animals through empirical research, behavioural monitoring, and evidence-based management practices that directly influence health and wellbeing.	visitor effects; behavioral monitoring	
Conservation and Ecology of Elephants and Rhinos	This cluster focuses on conservation status and threats facing elephants and rhinoceroses, including surveys, management strategies, anti-poaching, habitat loss, and human-wildlife conflict.	elephant conservation; rhino population; poaching mitigation; habitat assessment; human-wildlife conflict	28
Genetic Variation and Adaptation in Salmon	This cluster focuses on genetic diversity, population structure, and adaptive variation in Atlantic salmon using molecular and genomic approaches to inform conservation.	Atlantic salmon; genetic variation; MHC genes; population structure; conservation genetics	27
**Squirrel Disease Ecology and Conservation**	This cluster investigates impacts of infectious diseases on red squirrels, including squirrelpox virus, transmission dynamics involving invasive grey squirrels, and conservation implications. *Why wildlife health?:* Addresses the epidemiology, pathology, and conservation impacts of viral diseases such as squirrelpox and adenovirus in red and grey squirrels, with implications for disease management and species survival.	squirrelpox virus; red squirrel; grey squirrel; disease ecology; conservation biology	26
Extinct and extant Caribbean mammals	This cluster investigates evolutionary history, taxonomy, extinction, and conservation of Caribbean insular mammals using ancient DNA, morphometrics, palaeontology, and biogeography.	Caribbean mammals; insular extinction; ancient DNA; rodent taxonomy; island biogeography	25
**Bovine Tuberculosis and Badger Ecology**	This cluster focuses on epidemiology and control of bovine tuberculosis involving European badgers and cattle, addressing transmission, impacts of culling and vaccination, and policy evaluation. *Why wildlife health?:* Examines the epidemiology, transmission dynamics, and management of bovine tuberculosis	bovine tuberculosis; European badger; disease transmission; wildlife culling; epidemiology	23

Topic label	Description	Keywords	N papers
	involving European badgers as a wildlife reservoir, with consequences for livestock health and disease control.		
Population Dynamics and Conservation of Ungulates	This cluster investigates ecological and evolutionary drivers of ungulate population dynamics, including density dependence, harvesting practices, environmental variability, and management under change.	ungulates; population dynamics; harvesting impact; phenotypic plasticity; conservation management	21

Figures

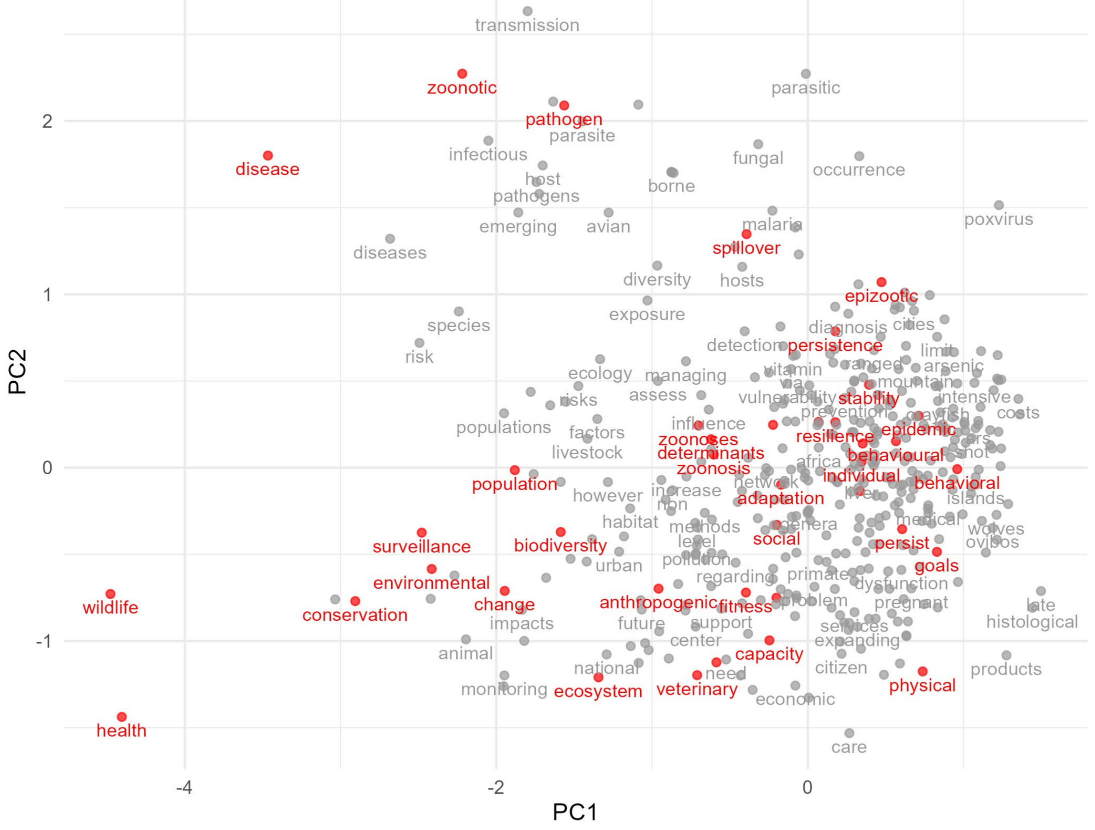

Figure S1. Two-dimensional semantic map of seed terms and their neighbours derived from the 100-dimensional GloVe embedding. Points represent terms projected onto the first two principal components, which together explain 9.2% of the variance. Seed terms are shown in red.

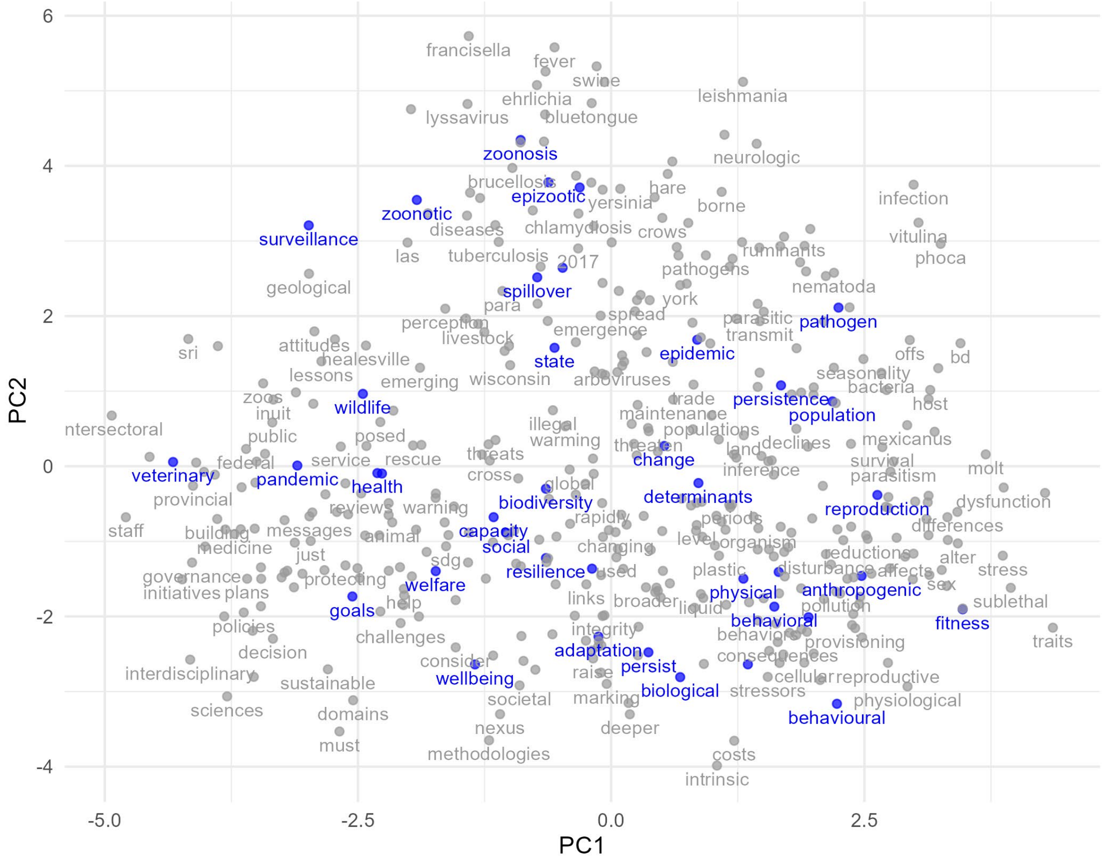

Figure S2. Two-dimensional semantic map of seed terms and their neighbours derived from the 100-dimensional word2vec skip-gram embedding. Points show terms projected onto the first two principal components, which together explain 9.7% of the variance. Seed terms are shown in blue.

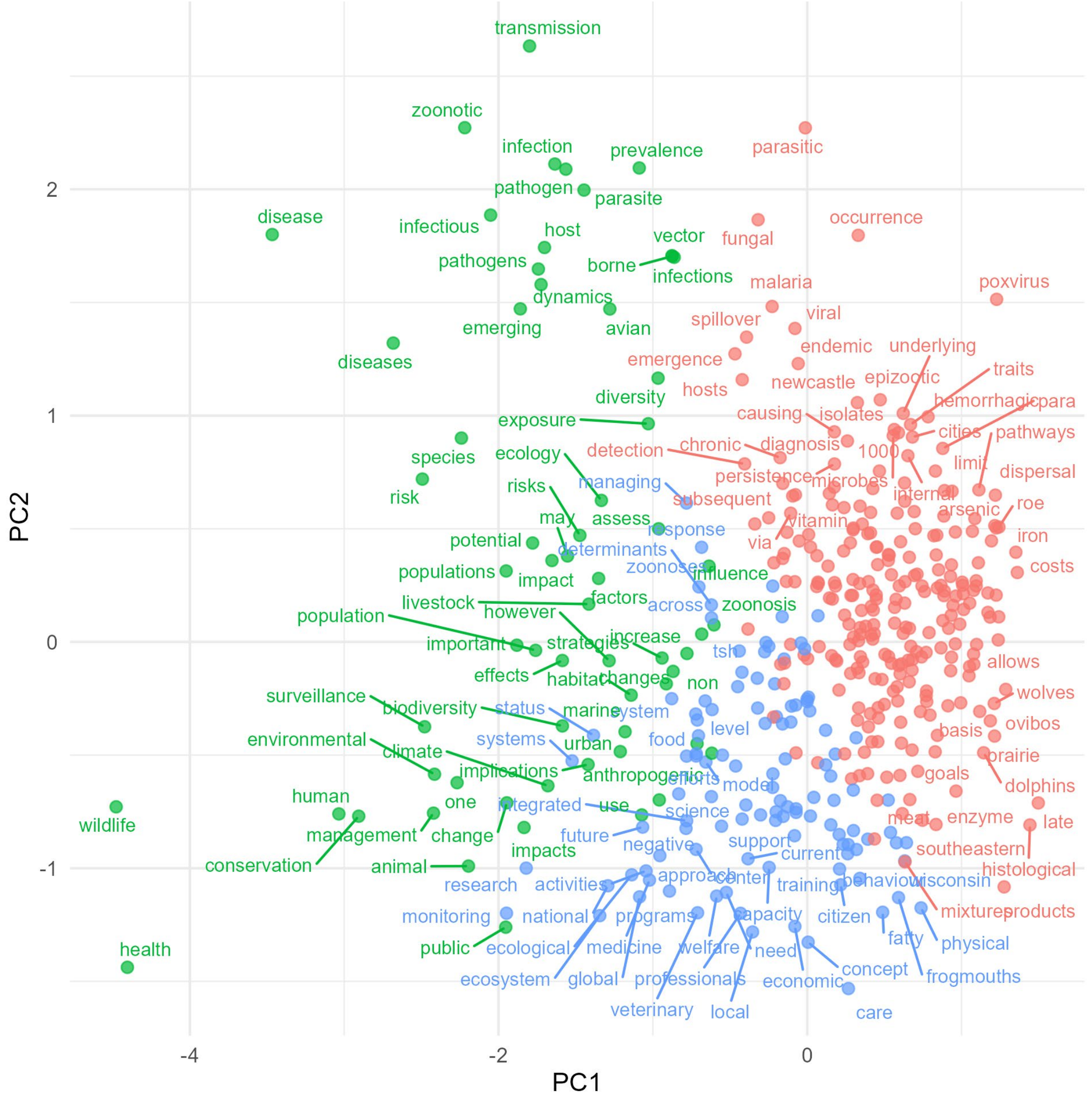

Figure S3. Two-dimensional semantic map of all clustered terms in the GloVe embedding space. Points represent terms projected onto the first two principal components (variance explained: 9.1%). Colours denote conceptual domains: green = Infection and disease ecology, blue = Monitoring, management, and policy, red = Ecological responses.

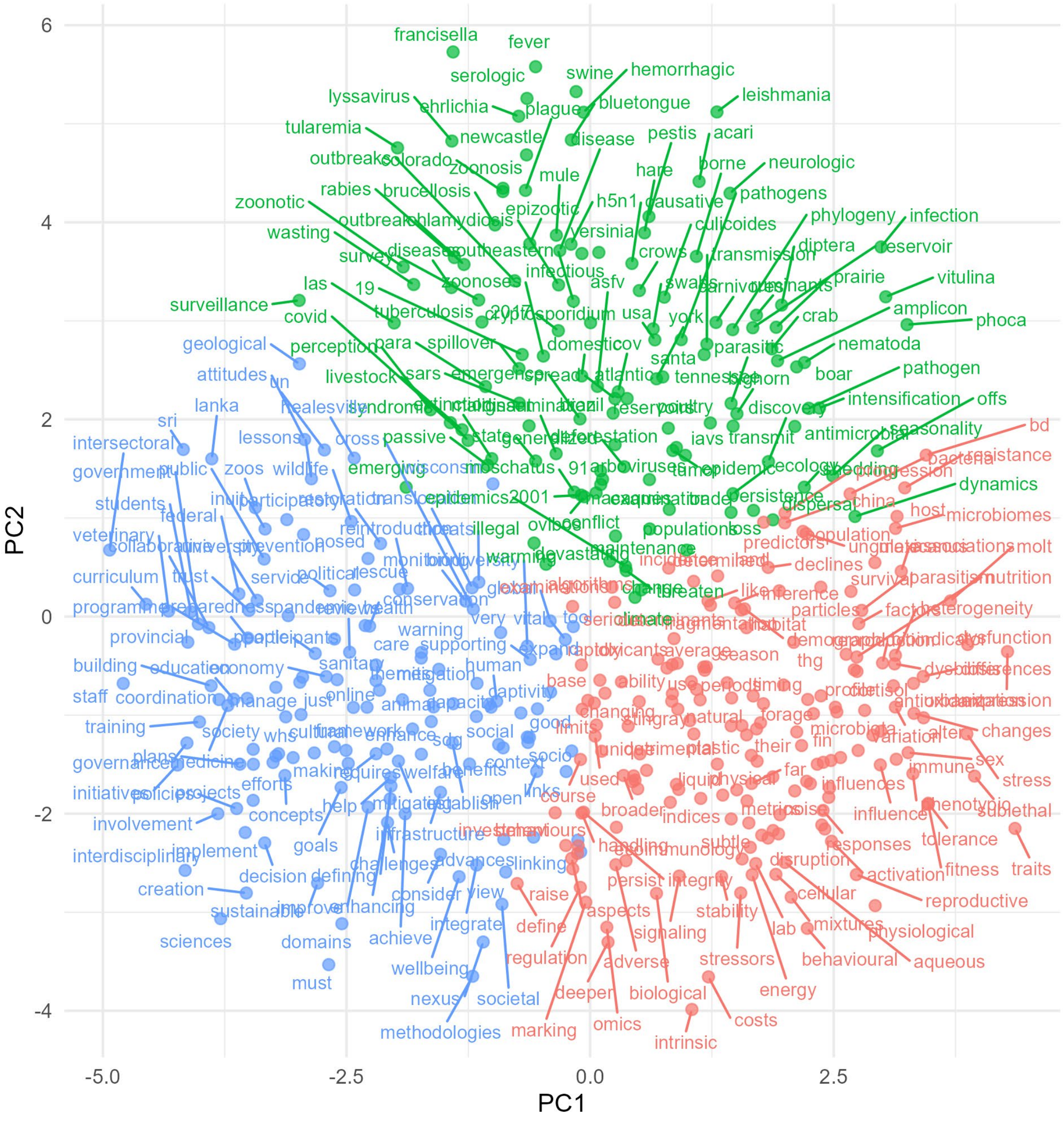

Figure S4. Two-dimensional semantic map of all clustered terms in the word2vec embedding space. Points represent terms projected onto the first two principal components (variance explained: 9.8%). Colours denote conceptual domains: green = Infection and disease ecology, blue = Monitoring, management, and policy, red = Ecological responses.

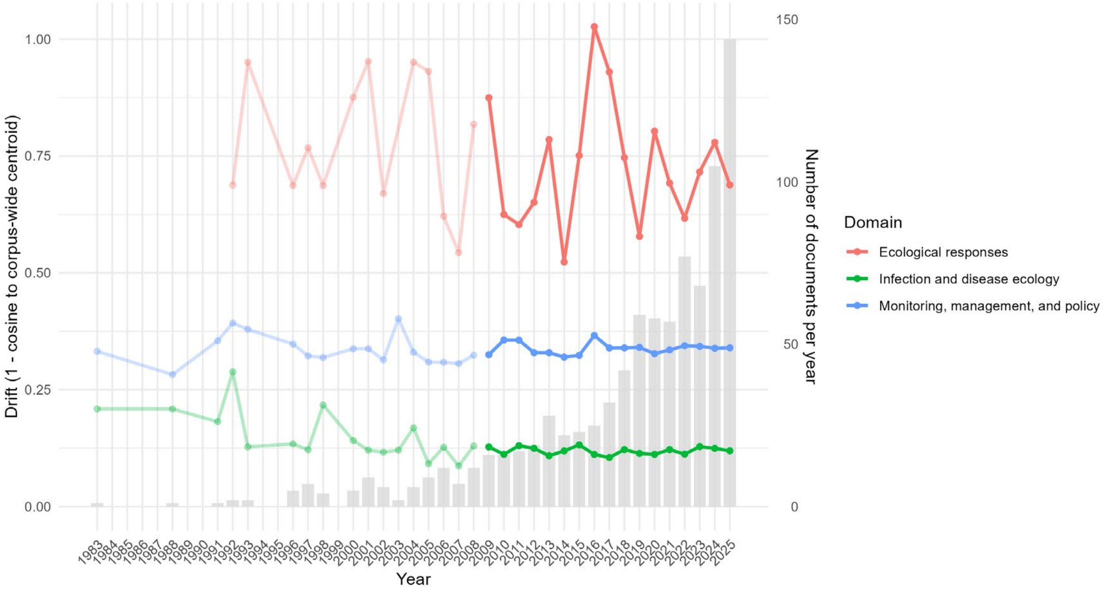

Figure S5. Semantic drift of conceptual domains from 2009 onwards. Lines show annual drift values for each domain relative to its corpus-wide centroid; bars show annual publication counts. Years with at least 15 documents are emphasised, reflecting greater robustness in centroid estimation.

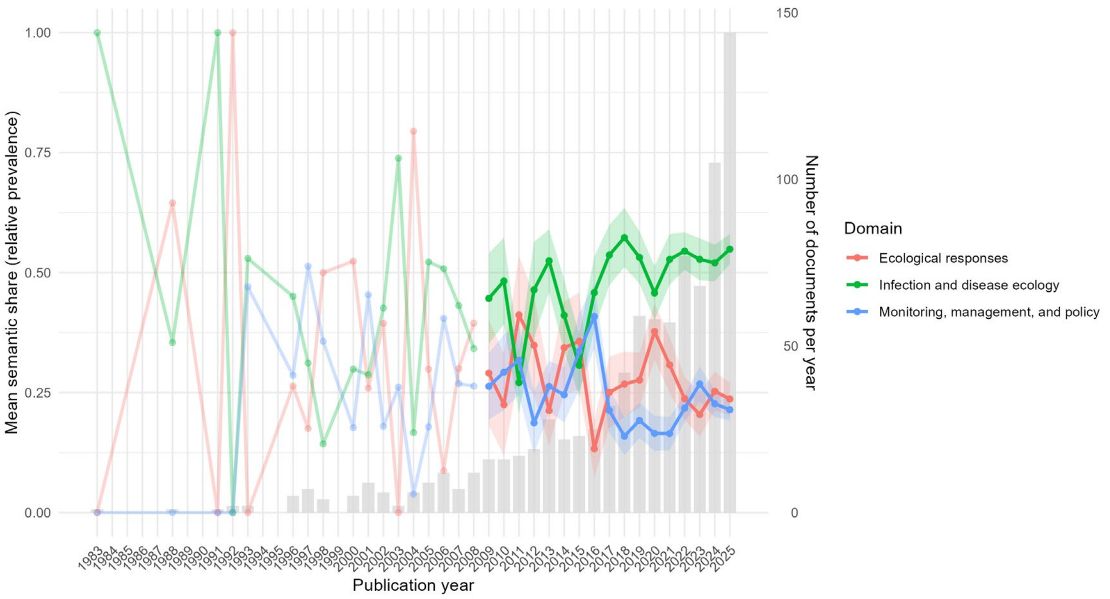

Figure S6. Temporal variation in the semantic share of conceptual domains. Lines show the mean within-document semantic share of each domain by publication year, derived from cosine similarity between TF–IDF-weighted document embeddings and fixed domain centroids in the GloVe embedding space. Shaded ribbons indicate ±1 standard error for years with at least 15 documents; years with fewer documents are shown with reduced opacity. Bars show annual publication counts.

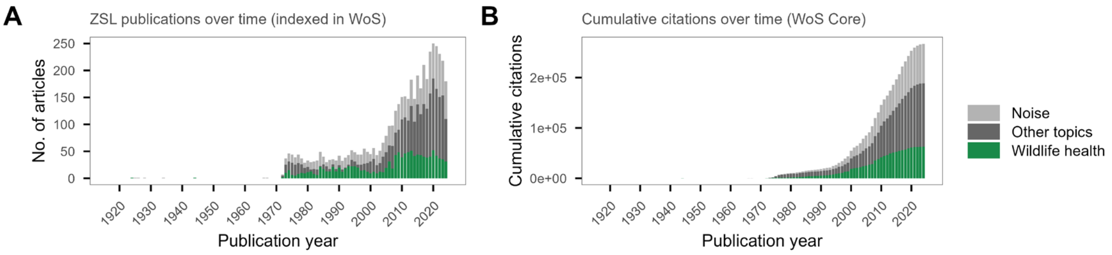

Figure S7. Temporal dynamics of ZSL research output. (A) Annual number of ZSL publications indexed in Web of Science, shown as stacked bars by high-level category: wildlife health, other topics, and noise. Counts are based on exact publication-year metadata and illustrate changes in absolute research output over time. (B) Cumulative citations over time for the same categories, calculated from Web of Science Core Collection citation counts and shown as stacked cumulative totals to visualise the historical accumulation of scholarly impact by category.
